# PTK2 is a potential biomarker and therapeutic target for EGFR- or TLRs-induced lung cancer progression via the regulation of the cross-talk between EGFR- and TLRs-mediated signals

**DOI:** 10.1186/s40364-024-00604-x

**Published:** 2024-05-31

**Authors:** Ji Young Kim, Ji Hye Shin, Mi-Jeong Kim, Bongkum Choi, Yeeun Kang, Jimin Choi, Seo Hyun Kim, Dohee Kwan, Duk-Hwan Kim, Eunyoung Chun, Ki-Young Lee

**Affiliations:** 1 Department of Immunology, Samsung Biomedical Research Institute, Sungkyunkwan University School of Medicine, 2066 Seobu-ro, Jangan-gu, Suwon, Gyeonggi-do 16419 Republic of Korea; 2 Department of Medicine, Sungkyunkwan University School of Medicine, Suwon, Republic of Korea; 3 Bioanalysis Center, GenNBio Inc., Seongnam, Republic of Korea; 4 Department of Metabiohealth, Sungkyun Convergence Institute, Sungkyunkwan University, Suwon, Republic of Korea; 5 Research and Development Center, CHA Vaccine Institute, 560 Dunchon-daero, Jungwon-gu, Seongnam-si, Gyeonggi-do 13230 Republic of Korea; 6 Department of Health Science and Technology, Samsung Medical Center, Samsung Advanced Institute for Health Science and Technology, Sungkyunkwan University School of Medicine, Seoul, Republic of Korea

**Keywords:** Protein tyrosine kinase 2, Epidermal growth factor receptor, Toll-like receptors, Non-small cell lung cancer, Defactinib

## Abstract

Protein tyrosine kinase 2 (PTK2), epidermal growth factor receptor (EGFR), and toll-like receptor (TLRs) are amplified in non-small cell lung cancer (NSCLC). However, the functional and clinical associations between them have not been elucidated yet in NSCLC. By using microarray data of non-small cell lung cancer (NSCLC) tumor tissues and matched normal tissues of 42 NSCLC patients, the genetic and clinical associations between PTK2, EGFR, and TLRs were analyzed in NSCLC patients. To verify the functional association, we generated *PTK2*-knockout (*PTK2*-KO) lung cancer cells by using CRISPR-Cas9 gene editing method, and performed in vitro cancer progression assay, including 3D tumor spheroid assay, and in vivo xenografted NSG (NOD/SCID/IL-2Rγ^null^) mouse assay. Finally, therapeutic effects targeted to PTK2 in lung cancer in response to EGF and TLR agonists were verified by using its inhibitor (Defactinib). In summary, we identified that up-regulated PTK2 might be a reliable marker for EGFR- or TLRs-induced lung cancer progression in NSCLC patients via the regulation of the cross-talk between EGFR- and TLRs-mediated signaling. This study provides a theoretical basis for the therapeutic intervention of PTK2 targeting EGFR- or TLRs-induced lung cancer progression.

**Supplementary Information:**

The online version contains supplementary material available at 10.1186/s40364-024-00604-x.

## To the editor

Accumulating evidence has demonstrated that the expression of PTK2, EGFR, or TLRs is associated with lung cancer [1–3]. Nevertheless, the functional and clinical associations between them in the regulation of lung cancer progression remain uncertain.

**Fig. 1:**
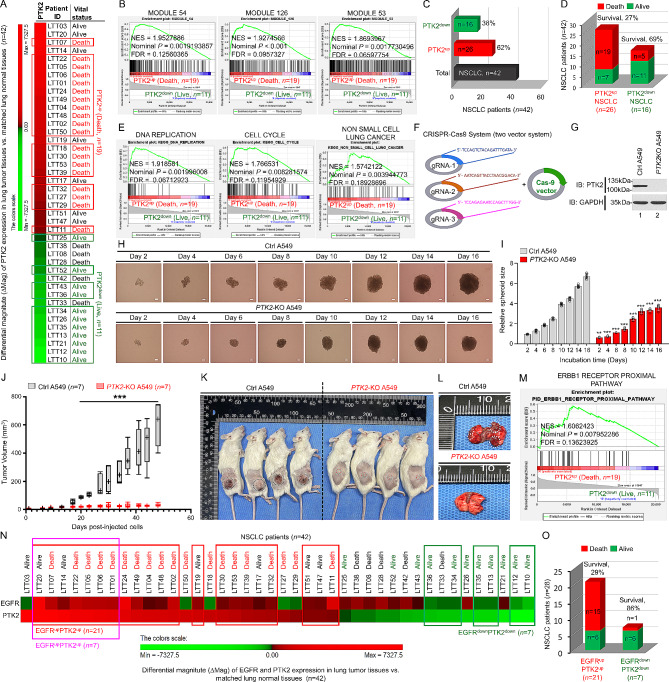
PTK2 expression associated with a poor prognosis and lung cancer progression. **A** Differential magnitude (ΔMag) of PTK2 between lung tumor tissues (LTTs) and matched lung normal tissues (mLNTs) was analyzed in 42 NSCLC patients. Among NSCLC patients (*n* = 42), PTK2^up^ NSCLC patients (*n* = 19) showed a poor prognosis as compared to PTK2^down^ NSCLC patients (*n* = 11). **B** Gene set enrichment analysis (GSEA, https://www.gsea-msigdb.org/gsea/index.jsp) was performed for PTK2^up^ (Death, *n* = 19) versus PTK2^down^ (Live, *n* = 11). Gene sets related to cancer modules are presented. NES, nominal P-value, and FDR q-values are indicated in the inner panel. **C and D** Based on the △Mag of PTK2 expression, NSCLC patients were divided into PTK2^up^ and PTK2^down^ NSCLC patients (**C**). Survival percent was analyzed in PTK2^up^ (*n* = 26) and PTK2^down^ (*n* = 16) NSCLC patients (**D**). **E** GSEA was performed for PTK2^up^ (Death, *n* = 19) versus PTK2^down^ (Live, *n* = 11). Gene sets related to cell cycle and NSCLC are presented. NES, nominal P-value, and FDR q-values are indicated in the inner panel. **F and G** Two-vector system was utilized to generate *PTK2*-KO lung cancer cells. Three gRNAs targeted to PTK2 were designed (**F**). *PTK2*-KO A549 cells were generated (**G**). **H and I** Ctrl A549 or *PTK2*-KO A549 cells were seeded and incubated at 37 °C for an additional 48 h to allow the formation of 3D spheroids in culture. The spheroid was incubated for different time periods as indicated. Spheroid formation and growth were evaluated using phase-contrast microscopy (scale bar, 100 μm) (**H**). The size of the spheroid was assessed using ImageJ Software. Error bars represent SD (*n* = 7) of three experiments (**I**). **, *P* < 0.01; ***, *P* < 0.001. **J-L** Ctrl A549 (5 × 10^6^ cells per mouse, *n* = 7) or *PTK2*-KO A549 cells (5 × 10^6^ cells per mouse, *n* = 7) were injected under NSG mice skin (back area). Tumor volume was measured with a caliper until 48 days after injection. Tumor volumes (mm^3^) were calculated as (length x width)^2^ × 0.5. Tumor growth curves are presented as average tumor volume ± SEM for each group in this study (**J**). Representative tumor-bearing NSG mice (*n* = 4) were shown at post-injection day 53. Tumors were indicated as black circles (Ctrl A549-injected NSG mice) or red circles (*PTK2*-KO A549-injected NSG mice) (**K**). A representative lung organ derived from NSG mice injected with Ctrl A549 or *PTK2*-KO A549 cells was shown. Metastatic tumors were indicated as white circles (**L**). ***, *P* < 0.001. (**M).** GSEA was performed for PTK2^up^ (Death, *n* = 19) versus PTK2^down^ (Live, *n* = 11). A gene set related to the EGFR-related pathway is presented. NES, nominal P-value, and FDR q-values are indicated in the inner panel. **N and O** Based on the △Mag of EGFR and PTK2 expression, NSCLC patients were divided into EGFR^up^PTK2^up^ (*n* = 21) and EGFR^down^PTK2^down^ (*n* = 7) NSCLC patients (**N**). Survival percent was analyzed in EGFR^up^PTK2^up^ (*n* = 21) and EGFR^down^PTK2^down^ (*n* = 7) NSCLC patients (**O**)

**Fig. 2:**
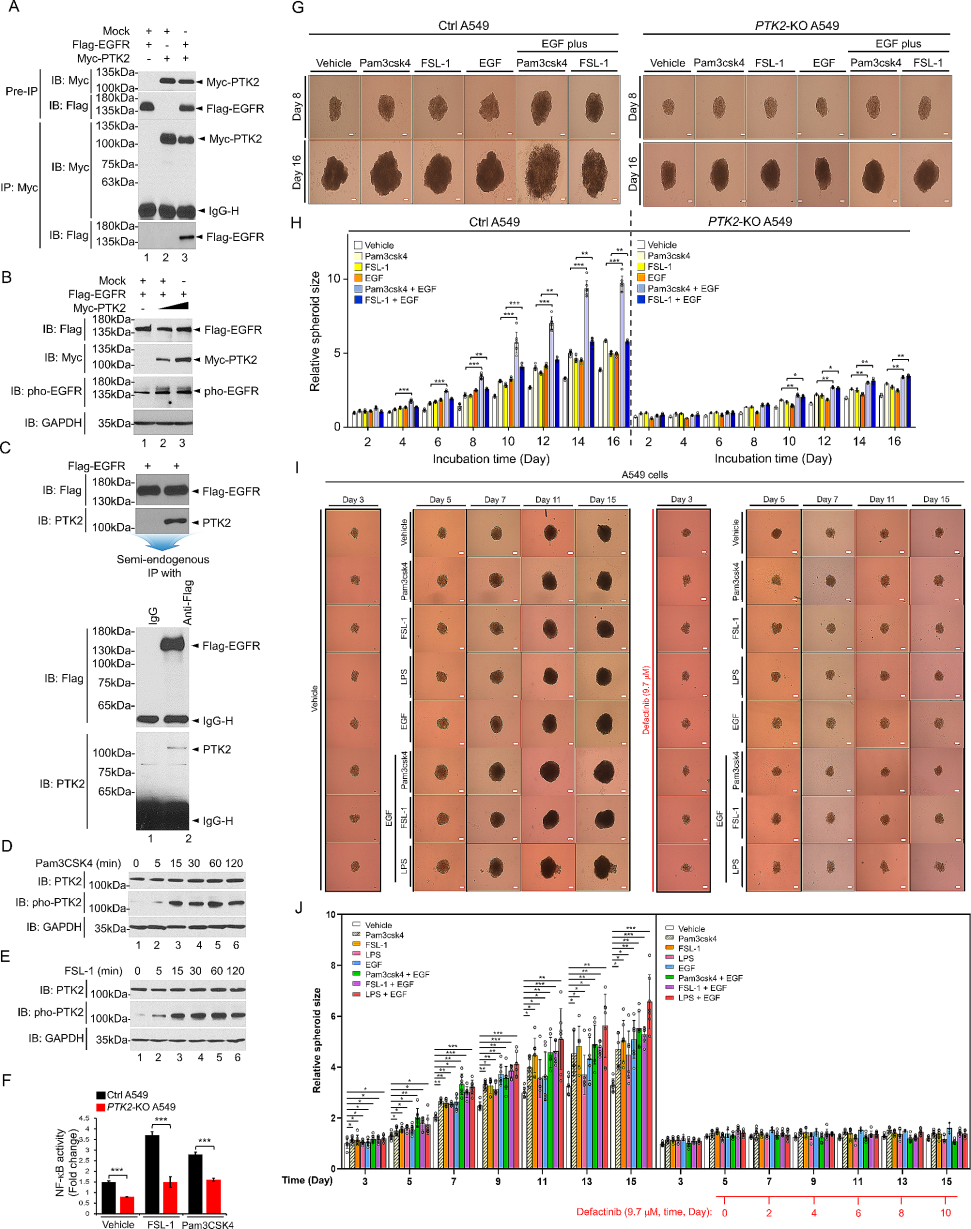
Biochemical mechanism study between PTK2 and EGFR or TLR signaling and therapeutic effects of Defactinib in 3D lung cancer spheroid formation in response to EGF and TLR agonists. **A** HEK-293T cells were transiently transfected with mock as a control vector, Flag-EGFR, or Myc-PTK2, as indicated. An immunoprecipitation assay was performed with anti-Myc antibody. Immunoblotting assay was then performed with anti-Flag or anti-Myc antibody. **B** A549 wild type (WT) cells were transiently transfected with mock as a control vector, Flag-EGFR, or different concentrations of Myc-PTK2, as indicated. An immunoblotting assay was performed with anti-Flag, anti-Myc, anti-pho-EGFR, and anti-GAPDH antibodies. **C** Semi-endogenous IP assay was performed in A549 wild type (WT) transiently transfected with Flag-EGFR. IP assay was performed with IgG as a control or anti-Flag antibody, and immune-blotting assay was performed with anti-Flag or anti-PTK2 antibody. **D and E** A549 cells were treated with vehicle, Pam3csk4 (**D**), or FSL-1 (**E**) for different periods, as indicated. An immunoblotting assay was performed with anti-PTK2, anti-pho-PTK2, and anti-GAPDH antibodies. **F** NF-κB dual-luciferase assay was performed for Ctrl A549 and *PTK2*-KO A549 cells treated with vehicle, FSL-1, or Pam3csk4, as indicated. Results are presented as mean ± SD of three independent experiments. ***, *P* < 0.001. **G** and **H** Ctrl A549 and *PTK2*-KO A549 cells were seeded into 96-well agarose-hydrogel plates and incubated for 2 days before treatments with TLR agonists or EGF. At post-cultured day 2 of spheroids, spheroids of Ctrl A549 and *PTK2*-KO A549 cells were treated with vehicle, Pam3csk4, FSL-1, EGF, Pam3csk4 plus EGF, or FSL-1 plus EGF for different periods. Spheroid formation and growth were evaluated using phase-contrast microscopy (scale bar, 100 μm). The full-day images were provided in Fig. S19. The size of the spheroid was assessed using ImageJ Software. Error bars represent SD (*n* = 5) of three experiments (**H**). *, *P* < 0.05; **, *P* < 0.01; ***, *P* < 0.001. **I and J**. As following protocol represented in Fig. S23A, the spheroids of A549 wild-type cells were treated with vehicle, Pam3csk4, FSL-1, LPS, EGF, Pam3csk4 plus EGF, FSL-1 plus EGF, or LPS plus EGF in the presence or absence of Defactinib (9.7 µM), as indicated. Spheroid formation and growth were evaluated using phase-contrast microscopy (scale bar, 100 μm) at different times, as indicated (**I**). The size of the spheroid was assessed using ImageJ Software. Error bars represent SD (*n* = 5) of three experiments (**J**). *, *P* < 0.05; **, *P* < 0.01; ***, *P* < 0.001

This study, as illustrated in Fig. S1, was designed to address the functional association of PTK2, EGFR, and TLRs in 42 NSCLC patients. We utilized the differential magnitude (ΔMag) analysis approach to stratify 42 NSCLC patients based on the expression levels of each gene in lung tumor tissues (*n* = 42) compared to matched lung normal tissues (*n* = 42). Subsequently, we calculated the survival rate based on the gene expression profile and patients’ clinical status. In the first association study between PTK2^up^ NSCLC patients (Death, *n* = 19) and PTK2^down^ NSCLC patients (Alive, *n* = 11), we found that gene sets related to cancer modules and cancer progression were highly enriched in PTK2^up^ NSCLC patients (Death, *n* = 19) (Fig. 1A; Table S1; Fig. S2A-Q; Fig. 1B; Fig. S3A-I), indicating that PTK2 expression is critically associated with cancer progression and poor survival rate in NSCLC patients (Fig. 1C, D). Importantly, gene sets related to cell cycle and NSCLC were significantly enriched in PTK2^up^ NSCLC patients (Death, *n* = 19) (Fig. 1E). To verify the PTK2 expression in lung cancer progression, PTK2-knockout (*PTK2*-KO) human lung cancer cells were generated by using CRISPR-Cas9 gene editing method (Fig. 1F, G; Fig. S4) [4–9]. The ability of cell proliferation, migration, and colony formation was significantly decreased in *PTK2*-KO A549 and *PTK2*-KO H1299 cells (Fig. S5A-J) [5–9]. Furthermore, 3D tumor spheroid formation assay revealed the marked attenuation of tumor spheroids in *PTK2*-KO A549 and *PTK2*-KO H1299 cells as compared to those of Ctrl A549 and Ctrl H1299 cells, respectively (Fig. 1H, I; Fig. S6A, B) [6, 10]. Notably, NSG mice xenografted with the *PTK2*-KO A549 cells showed a marked decrease in tumor growth (Fig. 1J, K) and metastasis into the lung tissues (Fig. 1L) as compared to NSG mice xenografted with the Ctrl A549 cells [11], strongly supporting the results of gene sets in PTK2^up^ NSCLC patients (Death, *n* = 19) vs. PTK2^down^ NSCLC patients (Alive, *n* = 11). Importantly, gene sets related to EGFR-associated pathways were enriched in PTK2^up^ NSCLC patients (Death, *n* = 19) (Fig. 1M; Fig. S7A-C). Therefore, we tried to assess the association between PTK2 and EGFR in NSCLC patients (2nd association study indicated in Fig. S1; Table S2; Fig. 1N). Patient survival was remarkably decreased in EGFR^up^PTK2^up^ NSCLC patients (Figs. 1O and 29% vs. 86%). GSEA revealed that gene sets related to cancer modules and cancer progression were highly enriched in PTK2^up^EGFR^up^ NSCLC patients (*n* = 7) vs. PTK2^down^EGFR^down^ NSCLC patients (*n* = 7) (Table S3; Fig. S8A-T; Fig. S9A-I). Moreover, gene sets related to FAK, EGFR-associated, and TOLL-associated pathways were highly enriched in PTK2^up^EGFR^up^ NSCLC patients (*n* = 7) (Fig. S10A-L), supposing that PTK2 may be functionally involved in the EGFR- and TLR-mediated signaling. To verify the functional association, we performed a biochemical assay. Interestingly, PTK2 interacted with EGFR and enhanced the activation of EGFR (Fig. 2A-C). Moreover, PTK2 interacted with TLR-mediated signaling molecules, such as TRAF6, IRAK1, and TAK1, and induced the activation of these proteins in responses to Pam3CSK4 (a TLR1/2 agonist) and FSL-1 (a TLR2/6 agonist) (Fig. S11A-J; Fig. 2D, E), leading to the activation of NF-κB in a PTK2-dependent manner (Fig. 2F, *PTK2*-KO A549 vs. Ctrl A549), suggesting that PTK2 positively regulates EGFR- and TLRs-mediated signaling for the activation of NF-κB (depicted in Fig. S11K).

Given the above results, we further tried to assess the association between them, PTK2, EGFR, TLR1, TLR2, and TLR6, in NSCLC patients (3rd association study indicated in Fig. S1; Table S4; Fig. S12A). Importantly, GSEA revealed that gene sets related to cancer modules and cancer progression were highly enriched in TLR1^up^TLR6^up^TLR2^up^PTK2^up^EGFR^up^ NSCLC patients (*n* = 5) vs. TLR1^down^TLR6^down^TLR2^down^PTK2^down^EGFR^down^ NSCLC patients (*n* = 4) (Fig. S12B-N; Fig. S13A-L). Furthermore, gene sets related to EGFR-associated pathways were significantly enriched in TLR1^up^TLR6^up^TLR2^up^PTK2^up^EGFR^up^ NSCLC patients (*n* = 5) (Fig. S14A-F). To verify the functional association between them, *PTK2*-KO A549 and *PTK2*-KO H1299 cells were treated with vehicle, Pam3CSK4, FSL-1, EGF, Pam3CSK4 plus EGF, FSL-1 plus EGF, and cancer progression assay and 3D tumor spheroid formation assay were performed. Interestingly, the ability of cell proliferation, migration, colony formation, and 3D spheroid formation were significantly attenuated in *PTK2*-KO A549 and *PTK2*-KO H1299 cells treated with vehicle, Pam3CSK4, FSL-1, EGF, Pam3CSK4 plus EGF, FSL-1 plus EGF, as compared to those of Ctrl A549 and Ctrl H1299 cells (Fig. S15A-H; Fig. S16A, B; Fig. S17A-H; Fig. S18A-F; Fig. S19; Fig. 2G, H), suggesting that PTK2 is functionally implicated in EGFR- and TLRs-mediated cancer progression. Having shown these results, we finally assessed whether PTK2 is a potential therapeutic target for the lung cancer progression induced by EGFR and TLRs. To do that, we used Defactinib, an inhibitor PTK2 [12], and performed a 3D tumor spheroid formation assay after the determination of IC_50_ in A549 and H1299 lung cancer cells (Fig. S20A, B; Fig. S21A, B). Notably, Defactinib effectively inhibited the 3D tumor spheroid formation of wild type A549 and H1299 cells in response to Pam3CSK4, FSL-1, LPS, EGF, Pam3CSK4 plus EGF, FSL-1 plus EGF, or LPS plus EGF as compared to those of the treatment of vehicle (Fig. S22A-C; Fig. S23A; Fig. 2I, J).

In summary, our results for the first time demonstrate that PTK2 expression is functionally associated with EGFR and TLRs in lung cancer progression (Fig. S23B), and the inhibition of PTK2 activity leads to the attenuation of lung cancer progression induced by EGF, TLRs, and EGF plus TLRs. We strongly believe that the current work can be a milestone in the field of precision cancer medicine developing valuable biomarkers targeted to EGFR or TLRs in lung cancer therapy.

### Electronic supplementary material

Below is the link to the electronic supplementary material.

Supplementary Material 1

Supplementary Material 2

Supplementary Material 3

Supplementary Material 4

Supplementary Material 5

## Data Availability

All data related to this article are shown or available upon request from the corresponding authors.

## Abbreviations

PTK2: Protein tyrosine kinase 2
NSCLC: Non-small cell lung cancer
EGFR: Epidermal growth factor receptor
TLR: Toll-like receptor
CRISPR-Cas9: Clustered regularly interspaced short palindromic repeats and CRISPR-associated protein 9
NSG: NOD scid gamma mouse
ΔMag: Differential magnitude
GSEA: Gene set enrichment assay
NES: Normalized enrichment score
FDR: False discovery rate
NF-κB: Nuclear factor kappa-light-chain-enhancer of activated B cells
IRAK: Interleukin-1 receptor-associated kinase
TRAF6: Tumor necrosis factor receptor associated factor 6
TAK1: Transforming growth factor-β-activated kinase 1
