# Supplementary material for: PTK2 is a potential biomarker and therapeutic target for EGFR- or TLRs-induced lung cancer progression via the regulation of the cross-talk between EGFR- and TLRs-mediated signals
Source: Biomark Res. 2024 May 31;12:52. doi: 10.1186/s40364-024-00604-x (PMC11141017; doi:10.1186/s40364-024-00604-x)
Supplement: Supplementary file 5 — Supplementary Material 5 [file 40364_2024_604_MOESM5_ESM.docx]

**Additional information**

**Materials and Methods**

**Patients and Samples**

This study was conducted in accordance with the ethical principles stated in the Declaration of Helsinki. It was approved by the Institutional Review Board (IRB#: 2010-07-204) of Samsung Medical Center (SMC, Seoul, Korea). Written informed consent to use pathological specimens for research was obtained from all patients before surgery. Clinical data including clinicopathologic features, molecular testing results, and treatment histories were obtained from clinical records (Table S1). Lung tumor tissues and matched lung normal tissues of NSCLC patients (*n* = 42, Table S1) who had been diagnosed with lung cancer stage IA to stage IIIA were obtained from SMC. Lung tumor and matched normal specimens of enrolled patients were immediately frozen in liquid nitrogen and stored at −80 °C until use. Lung tumor tissues and matched lung normal tissues were verified by the Department of Laboratory Medicine and Pathology at SMC.

**Xenografted NSG mouse model**

NOD/SCID/IL-2Rγnull (NSG) mice were purchased from the Jackson Laboratory (Bar Harbor, ME, USA) and maintained under specific pathogen-free conditions in accordance with ethical guidelines for the care of these mice at the Bioanalysis Center Animal Facility, GenNBio Inc. (Seongnam, Korea). All experimental procedures were approved by the Institutional Animal Care and Use Committee (IACUC) of the Bioanalysis Center Animal Facility (IACUC #: 23-10-01). NSG mice at 6–8 weeks old were used to generate xenografted NSG mice. Control (Ctrl) A549 (5 × 10^6^ cells per mouse, *n* = 7) or *PTK2*-knockout (KO) A549 cells (5 × 10^6^ cells per mouse, *n* = 7) in serum-free RPMI were injected under NSG mice skin (back area) [1]. The final injection volume was 100 μL/mouse containing a 1:1 v/v mixture of ice-chilled Matrigel (BD Biosciences, La Jolla, CA, USA), which was kept on ice until injection. After injecting cancer cells, tumor volume was measured with a caliper until 53 days after injection. Tumor volumes (mm^3^) were calculated as (length x width)^2^ × 0.5. Tumor growth curves are presented as average tumor volume ± SEM for each group in this study. All studies involving mice were approved by the Nemours IACUC.

**Cells**

A549 cells (human lung cancer cell line; CCL-185, American type culture collection (ATCC), Manassas, VA, USA) and H1299 cells (human non-small cell lung cancer cell line; CRL-5803, ATCC) were maintained in RPMI 1640 medium (LM011-01, Welgene, Daegu, Korea) supplemented with 10% fetal bovine serum (FBS), penicillin (100 μg/mL), and streptomycin (100 μg/mL) in a 5% CO2 humidified atmosphere at 37 °C. Human embryonic kidney (HEK) 293T cells (ATCC, CRL-11268) were cultured and maintained in Dulbecco's modified Eagle's medium (DMEM; LM001-05, Welgene, Daegu, Korea) with 10% FBS.

**Antibodies and Reagents**

Anti-PTK2 (3285S), anti-Flag (F3165), anti-pho-EGFR (2236S), anti-pho-TAK1 (9339S), and anti-pho-PTK2 (8556S) antibodies were purchased from Cell Signaling Technology (Danvers, MA, USA). Anti-GAPDH (sc-47724), anti-Myc (sc40), anti-IRAK1 (sc-5288), anti-Ub (sc-8017), and anti-TAK1 (sc-7967) antibodies were purchased from Santa Cruz Biotechnology (Santa Cruz, CA, USA). Pam3CysSerLys4 (Pam3csk4, tlrl-pms) and Pam2CGDPKHPKSF (FSL-1, tlrl-fsl) were purchased from InvivoGen (San Diego, CA, USA). Goat anti-rabbit IgG (HRP) (GTX213110-01) antibody was purchased from GeneTex Inc. (Irvine, CA, USA). Rabbit anti-mouse IgG H&L (HRP) (ab6728) antibody was purchased from Abcam (Cambridge, MA, USA). TrueBlot® secondary antibodies (18-8816-33, 18-8817-33) were purchased from Cambridge Bioscience (Cambridge, UK). Lipopolysaccharide (LPS; L3024), dimethyl sulfoxide (DMSO; D4540), Dulbecco’s phosphate-buffered saline (DPBS; D8537), glutaraldehyde (G6257-100ml), crystal violet (C6158-50g), EGF (SRP3027), and Thiazolyl Blue Tetrazolium Bromide (MTT; M5655) were purchased from Sigma-Aldrich (St. Louis, MO, USA). Lipofectamine 2000 (11668019) was purchased from Thermo Fisher Scientific (Waltham, MA, USA). Defactinib (HY-12289A) was purchased from MedChemExpress (Monmouth Junction, NJ, USA).

**Plasmid constructs**

pWZL Neo Myr Flag PTK2 (20610), W118-1_Flag-hIRAK1 (180405), Flag-TRAF6 (21624), and pRK6-HA-TAK1 (44160) vectors were purchased from Addgene (Watertown, MA, USA). pCMV-3Tag-7 (240202) and pCMV-3Tag-6 (240200) vectors were purchased from Agilent technologies (Santa Clara, CA, USA). Using pWZL Neo Myr Flag PTK2 plasmid, full-length PTK2 was cloned into the pCMV-3Tag-6 vector or pCMV-3Tag-7 vector to generate Flag-PTK2 vector or Myc-PTK2 vector, respectively. Using Flag-hIRAK1 plasmid, full-length IRAK1 was cloned into pCMV-3Tag-7 vector to generate Myc-IRAK1. Using pRK6-HA-TAK1 plasmid, full-length TAK1 was cloned into pCMV-3Tag-6 vector to generate Flag-TAK1 vector. Truncated mutants of Flag-TRAF6, Flag-TRAF6 110–522, Flag-TRAF6 260–522, and Flag-TRAF6 349-522 were generated as previously described [24].

**Generation of PTK2-Knockout (*PTK2*-KO) Cell Line with CRISPR/Cas9**

To generate *PTK2*-KO lung cancer cells with CRISPR/Cas9 gene editing method, we used two vector systems, including single guide RNA (sgRNA) and CRISPR-associated protein 9 (Cas9) vectors, as previously described [25-30]. sgRNA and Cas9 vectors were kindly provided by Dr. Daesik Kim (Sungkyunkwan University School of Medicine, Suwon, Korea). Guide RNA sequences for CRISPR/Cas9 were designed on the CRISPR design website (http://crispr.mit.edu/) provided by the Feng Zhang Lab. Insert oligonucleotides for human PTK2 gRNA were: 5’-TCCAGTCTACAGATTTGATA-3’ (gRNA-1) / 5’-AATCAGTTACCTAACGGACA-3’ (gRNA-2) / 5’-TCCAGAGAATCCAGCTTTGG-3’ (gRNA-3). Complementary oligonucleotides to guide RNAs (gRNAs) were annealed and cloned into a sgRNA vector. sgRNA vectors expressing gRNA of PTK2 and Cas9 vector expressing Cas9 were transfected into A549 cells and H1299 cells using Lipofectamine 2000 (Thermo Fisher Scientific, Waltham, MA, USA) according to the manufacturer’s instructions. After two weeks, colonies were isolated from 96-well plates and expression levels of PTK2 were analyzed with western blotting.

**Western Blotting (WB) Assay**

Control (Ctrl) A549 lung cancer cells were seeded into 12-well plates and cultured. Cells were treated with vehicle (DMSO, 0.1 % v/v concentration), Pam3csk4 (3 μg/mL), or FSL-1 (300 ng/mL) for different periods. After collecting cells, cell lysates were separated by sodium dodecyl sulfate-polyacrylamide gel electrophoresis (SDS-PAGE, 8~12 %) and immune-probed with anti-PTK2, anti-pho-PTK2, and anti-GAPDH (as loading control) antibodies.

**Immunoprecipitation (IP) Assay**

HEK-293T cells were transiently transfected with mock (a relevant control vector), Flag-TRAF6, Flag-TRAF6 truncated mutants, Myc-PTK2, Flag-PTK2, Myc-IRAK1, or Flag-TAK1 as indicated in each figure for 24 hr. After collecting cells, cell lysates were prepared and immunoprecipitated with anti-Flag or anti-Myc antibody. IP complexes were separated by sodium dodecyl sulfate-polyacrylamide gel electrophoresis (SDS-PAGE, 8–12%) and immune-probed with anti-Myc or anti-Flag antibody. For semi-endogenous IP, H1299 wild type (WT) cells were transiently transfected with mock or Flag-PTK2 for 24 hr and then stimulated with vehicle or Pam3csk4 (7 μg/mL) for 6 hr. After collecting cells, cell lysates were prepared and immunoprecipitated with anti-IgG or anti-Flag antibody. IP complexes were separated by sodium dodecyl sulfate-polyacrylamide gel electrophoresis (SDS-PAGE, 8–12%) and immune-probed with anti-TAK1, anti-IRAK1, or anti-Flag antibody.

**Ubiquitination Assay**

HEK-293T cells were transiently transfected with mock (a relevant control vector), Flag-TRAF6, Myc-PTK2, or Flag-TAK1 for 24 hr. After collecting cells, cell lysates were prepared and immunoprecipitated with anti-Flag antibody. IP complexes were separated by sodium dodecyl sulfate-polyacrylamide gel electrophoresis (SDS-PAGE, 8–12%) and immune-probed with anti-Myc, or anti-Flag antibody.

**Wound-Healing Migration Assay**

A wound-healing migration assay was performed following previous protocols [2-6]. Briefly, Control (Ctrl) A549, *PTK2*-KO A549, Ctrl H1299, and *PTK2*-KO H1299 cells were seeded into 12-well plates and cultured to reach confluence. Cell monolayers were gently scratched and washed with a culture medium. After floating cells and debris were removed, cells attached to culture plates were treated with vehicle (DMSO, 0.1% v/v concentration), Pam3csk4 (3 μg/mL), FSL-1 (300 ng/mL), EGF (20 ng/mL), Pam3csk4 (3 μg/mL) plus EGF (20 ng/mL), or FSL-1 (300 ng/mL) plus EGF (20 ng/mL) for different time periods. Cell images were captured after culturing for different periods as indicated in each experiment.

**Transwell Migration Assay**

Control (Ctrl) A549, *PTK2*-KO A549, Ctrl H1299, and *PTK2*-KO H1299 lung cancer cells were suspended in a culture medium (250 μL) and added to the upper compartment of a 24-well Transwell® chamber (8 μm pore; Corning, 3422). Ctrl A549, Ctrl H1299, *PTK2*-KO A549, or *PTK2*-KO H1299 lung cancer cells and culture medium (250 μL) were mixed with vehicle (DMSO, 0.1 % v/v concentration), Pam3csk4 (3 μg/mL), FSL-1 (300 ng/mL), EGF (20 ng/mL), Pam3csk4 (3 μg/mL) plus EGF (20 ng/mL), or FSL-1 (300 ng/mL) plus EGF (20 ng/mL) and incubated at 37 °C for 24 h. Migratory cells would pass through polycarbonate membranes and cling to the bottom side. Non-migratory cells would stay in the upper chamber. After removing non-migratory cells, migratory cells were fixed using 2.5 % glutaraldehyde (Sigma-Aldrich, G6257-100 mL) and then stained with 0.1% crystal violet (Sigma-Aldrich, C6158-50g).

**Anchorage-Independent Soft Agar Colony Formation Assay**

Anchorage-independent soft agar colony formation assay was performed following previous protocols [2-6]. Briefly, Ctrl A549, *PTK2*-KO A549, Ctrl H1299, and *PTK2*-KO H1299 cells (1 × 10^4^ cells /well) mixed with 0.3% agarose (Biotechnology Grade, GA001.500) in complete medium were plated onto the bottom of a 0.5% agar layer in a 6-well plate with a complete medium. Growth medium (2 mL) with vehicle (DMSO, 0.1 % v/v concentration), Pam3csk4 (1 μg/mL), FSL-1 (100 ng/mL), EGF (10 ng/mL), Pam3csk4 (1 μg/mL) plus EGF (10 ng/mL), or FSL-1 (100 ng/mL) plus EGF (10 ng/mL) was added on top of the layer and cells were incubated at 37 °C for 42 days.

**Colony Formation Assay**

The ability of a single cell to grow into a colony was determined with a colony formation assay as previously described [2-7]. Ctrl A549, *PTK2*-KO A549, Ctrl H1299, and *PTK2*-KO H1299 cells were harvested with trypsin-EDTA and resuspended in a singular form. Then 700 cells/well were plated into a 6-well plate and treated with a vehicle (DMSO, 0.1% v/v concentration), Pam3csk4 (2 μg/mL), FSL-1 (200 ng/mL), EGF (20 ng/mL), Pam3csk4 (2 μg/mL) plus EGF (20 ng/mL), or FSL-1 (200 ng/mL) plus EGF (20 ng/mL). After incubation for 8 or 10 days, colonies were stained with 0.5% crystal violet (Sigma-Aldrich, C6158-50g) for 30 min at room temperature.

**Three-Dimension (3D) Spheroids Formation Assay Using Agarose-Coated Plates**

3D Spheroids formation assay was performed following previous protocols [8, 9]. Briefly, 1.5% agarose hydrogel was added to each well of a 96-well culture plate and incubated at room temperature (RT) for 30 min. Ctrl A549, *PTK2*-KO A549, Ctrl H1299, and *PTK2*-KO H1299 cells were seeded into 100 µl growth medium at a concentration of 125 or 500 cells per well. Plates were incubated at 37 °C for an additional 48 hours to allow the formation of 3D spheroids in culture. The spheroid was added with vehicle (DMSO, 0.1% v/v concentration), Pam3csk4 (2 μg/mL), FSL-1 (200 ng/mL), EGF (10 ng/mL), Pam3csk4 (2 μg/mL) plus EGF (10 ng/mL), or FSL-1 (200 ng/mL) plus EGF (10 ng/mL) and incubated for additional time periods. Spheroid formation and growth were evaluated using phase-contrast microscopy. The size of the spheroid was assessed using ImageJ Software (National Institutes of Health, Bethesda, MD, USA). To determine IC50 value of Defactinib in wild type A549 or H1299 cells, 3D Spheroids of A549 or H1299 wild type cells were treated with vehicle (DMSO, 0.1% v/v concentration) or different concentrations of Defactinib (30 μM to 3 nM/mL). Tumor spheroids were incubated for different times, and spheroid formation and growth were evaluated using phase-contrast microscopy. The size of the spheroid was assessed using ImageJ Software (National Institutes of Health, Bethesda, MD, USA). IC50 value was calculated by Prism (GraphPad Software, Inc.). To evaluate therapeutic effects of Defactinib, 3D Spheroids of A549 or H1299 wild type cells were treated with vehicle (DMSO, 0.1% v/v concentration), Pam3csk4 (3 μg/mL), FSL-1 (300 ng/mL), LPS (10 μg/mL), EGF (10 ng/mL), Pam3csk4 (3 μg/mL) plus EGF (10 ng/mL), FSL-1 (300 ng/mL) plus EGF (10 ng/mL), or LPS (10 μg/mL) plus EGF (10 ng/mL) in the presence or absence of Defactinib (9.7 μM, A549 cells; 1.5 μM, H1299 cells), and incubated for additional time periods. Spheroid formation and growth were evaluated using phase-contrast microscopy. The size of the spheroid was assessed using ImageJ Software (National Institutes of Health, Bethesda, MD, USA).

**MTT Assay**

Ctrl A549, *PTK2*-KO A549, Ctrl H1299, and *PTK2*-KO H1299 cells were seeded into 96-well culture plates at a density of 500 or 700 cells/well, treated with vehicle (DMSO, 0.1% v/v concentration), Pam3csk4 (1.5 μg/mL), FSL-1 (150 ng/mL), EGF (15 ng/mL), Pam3csk4 (1.5 μg/mL) plus EGF (15 ng/mL), or FSL-1 (150 ng/mL) plus EGF (15 ng/mL) and grown in a culture medium supplemented with 10% FBS for different time periods. Cell viability was measured using an MTT reagent (Sigma-Aldrich, M5655) dissolved in PBS (1 mg/mL). On the day when measurements were taken, the medium was carefully replaced on fresh RPMI + 10% FBS with diluted MTT (1:10, 10% MTT) and incubated for 3 h at 37 °C. After removing the incubation medium, formazan crystals were dissolved in a 100 μl solution of DMSO. MTT reduction was quantified by measuring the absorbance at 595 nm using a Bio-Rad Model 680 microplate reader (Bio-Rad, CA, USA). Each test was repeated at least four times in quadruplicate.

**NF-κB Luciferase Reporter Assay**

Luciferase reporter assay was performed as previously described [3]. Briefly, Ctrl A549, *PTK2*-KO A549, Ctrl H1299, and *PTK2*-KO H1299 cells were transfected with mock, pBIIx-luc NF-κB-dependent reporter constructs, or Renilla luciferase vector (Promega, Madison, WI, USA). At 24 h post-transfection, cells were treated with vehicle (DMSO, 0.1% v/v concentration), Pam3csk4 (4 μg/mL), or FSL-1 (400 ng/mL) for 24 h and lysed. Luciferase activity was measured using a dual luciferase assay kit (Promega, 72050).

**Microarray Analysis**

Microarray analysis was performed as previously described [10-12]. Total RNAs were extracted from tumor and matched normal tissues of 42 patients with NSCLC with Trizol (Thermo Fisher Scientific, 15596026) and purified using RNeasy columns (Qiagen, 74106) according to each manufacturer’s protocol.

**Gene Set Enrichment Analysis (GSEA)**

Different magnitudes (∆ Mags) of PTK2 and EGFR expression were obtained from pre-processed microarray data between lung tumor tissues and matched lung normal tissues. Patients (*n* = 19) with up-regulated PTK2, patients (*n* = 11) with down-regulated PTK2, patients (*n* = 7) with up-regulated PTK2 and EGFR, and patients (*n* = 7) with down-regulated PTK2 and EGFR, patients (*n*=5) with up-regulated TLR1, TLR6, TLR2, EGFR, and PTK2, patients (*n*=4) with down-regulated TLR1, TLR6, TLR2, EGFR, and PTK2 were selected based on different magnitudes of PTK2 and EGFR. To identify genes showing significant differences, normalized enrichment score (NES), nominal P-value, false discovery rate (FDR), and q-values were analyzed by GSEA (http://www.gsea-msigdb.org/gsea/index.jsp).

**Human Protein Atlas (HPA) Analysis**

Expression level of PTK2 in normal and lung cancer types, lung adenocarcinoma, and lung squamous cell carcinoma were analyzed using The Human Protein Atlas (HPA) (https://www.proteinatlas.org/).

**TCGA Data Analysis**

Overall survival of LUAD and LUSC according to the expression of PTK2 was analyzed using TCGA data (GEPIA, gene expression profiling interactive analysis; http://gepia.cancer-pku.cn/detail.php?gene=PTK2).

**Statistical Analysis**

All data are expressed as mean ± SD (standard deviation). Statistical significance was determined by Student’s t-test using GraphPad Prism 5.0 (GraphPad Software, San Diego, CA, USA). P-values are marked by asterisks (*, P < 0.05; **, P < 0.01; and ***, P < 0.001).

**References**

1 Kim MJ, Choi B, Kim JY, et al. USP8 regulates liver cancer progression via the inhibition of TRAF6-mediated signal for NF-κB activation and autophagy induction by TLR4. Transl Oncol. 2022;15(1):101250.

2 Kim JY, Shin JH, Kim MJ, et al. β-arrestin 2 negatively regulates lung cancer progression by inhibiting the TRAF6 signaling axis for NF-κB activation and autophagy induced by TLR3 and TLR4. Cell Death Dis. 2023;14(7):422.

3 Kim MJ, Kim JY, Shin JH, et al. FFAR2 antagonizes TLR2- and TLR3-induced lung cancer progression via the inhibition of AMPK-TAK1 signaling axis for the activation of NF-κB. Cell Biosci. 2023;13(1):102.

4 Kim MJ, Kim JY, Shin JH, et al. The SARS-CoV-2 spike protein induces lung cancer migration and invasion in a TLR2-dependent manner. Cancer Commun (Lond). 2023 Sep 13.

5 Kim JY, Kim MJ, Lee JS, et al. Stratifin (SFN) regulates lung cancer progression via nucleating the Vps34-BECN1-TRAF6 complex for autophagy induction. Clin Transl Med. 2022;12(6):e896.

6 Kim MJ, Lee JS, Kim JY, et al. CRBN is downregulated in lung cancer and negatively regulates TLR2, 4 and 7 stimulation in lung cancer cells. Clin Transl Med. 2022;12(9):e1050.

7 Cong L, Ran FA, Cox D, et al. Multiplex genome engineering using CRISPR/Cas systems. Science. 2013;339(6121):819-23.

8 Shin JH, Kim MJ, Kim JY, et al. CXCR5 and TLR4 signals synergistically enhance non-small cell lung cancer progression. Clin Transl Med. 2024;14(1):e1547.

9 Zanoni M, Piccinini F, Arienti C, et al. 3D tumor spheroid models for in vitro therapeutic screening: a systematic approach to enhance the biological relevance of data obtained. Sci Rep. 2016;6:19103.

10 Kim MJ, Min Y, Son J, et al. AMPKα1 Regulates Lung and Breast Cancer Progression by Regulating TLR4-Mediated TRAF6-BECN1 Signaling Axis. Cancers (Basel). 2020;12(11):3289.

11 Min Y, Wi SM, Kang JA, et al. Cereblon negatively regulates TLR4 signaling through the attenuation of ubiquitination of TRAF6. Cell Death Dis. 2016 Jul 28;7(7):e2313.

12 Kim Y, Lee BB, Kim D, et al. Clinicopathological Significance of RUNX1 in Non-Small Cell Lung Cancer. J Clin Med. 2020;9(6):1694.

**Supplementary figure legends**

**
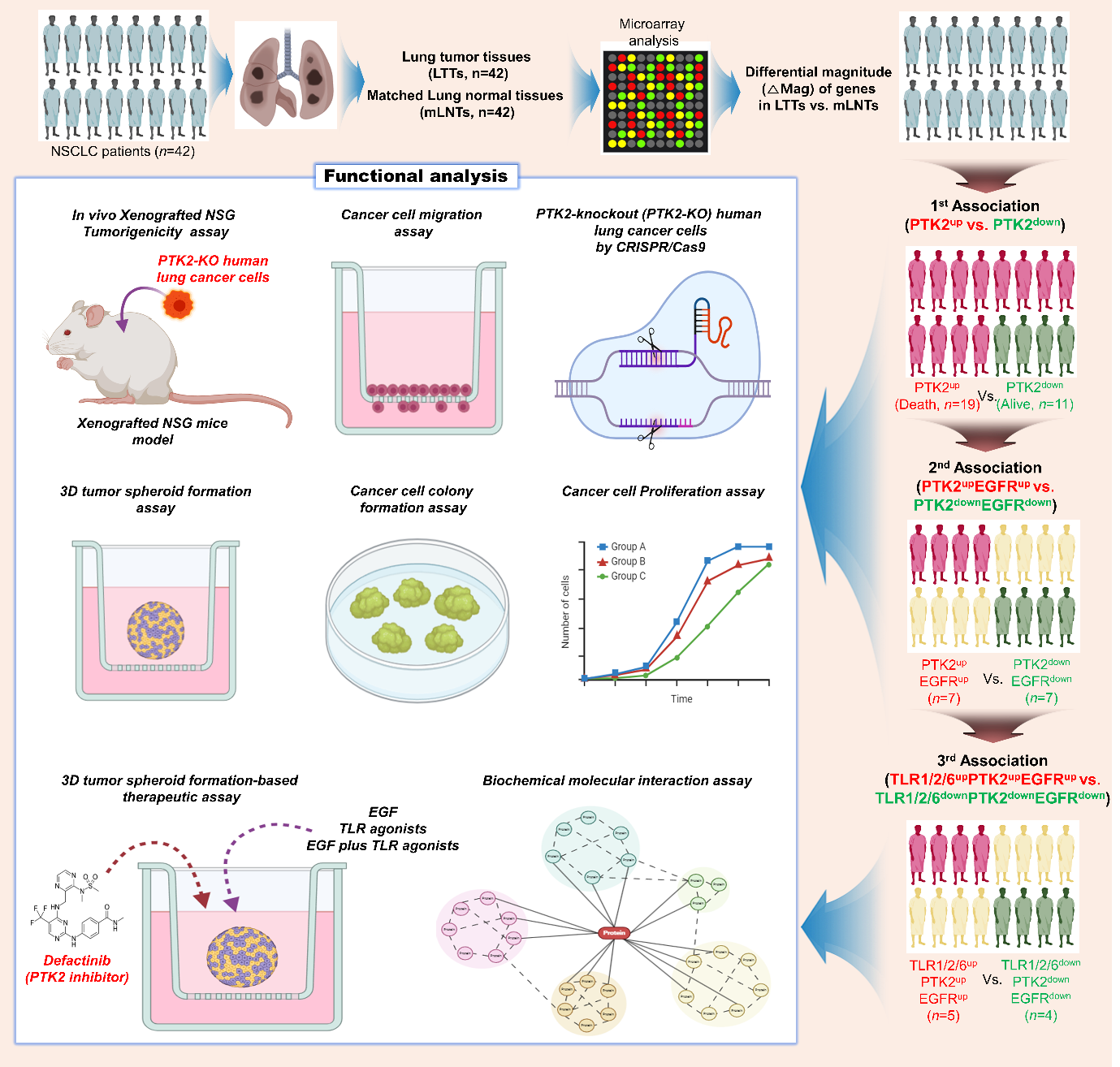
**

**Fig. S1** The experimental design in this study.


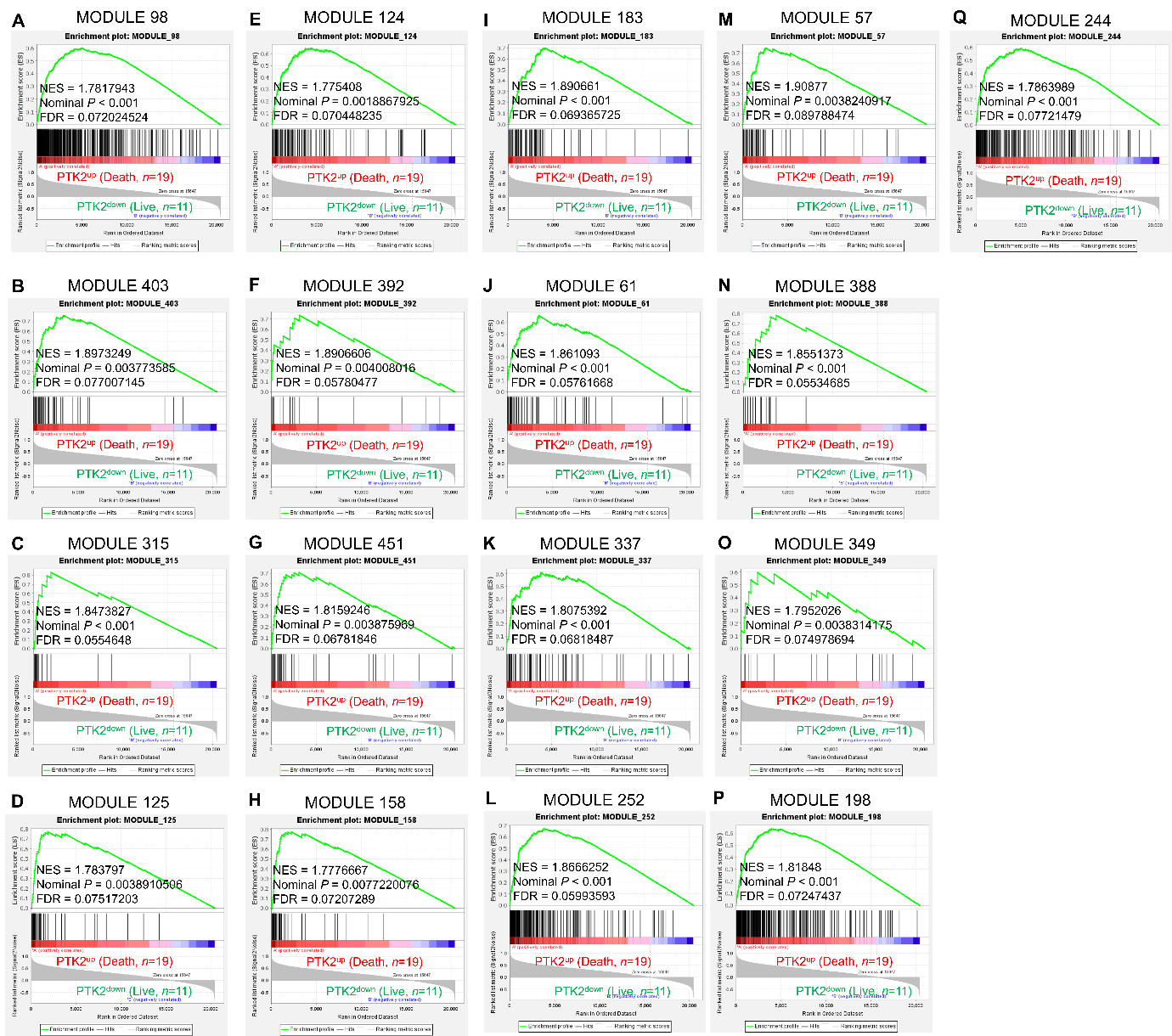


**Fig. S2 A-Q** Gene set enrichment analysis (GSEA, https://www.gsea-msigdb.org/gsea/index.jsp) was performed for PTK2^up^ (Death, *n*=19) versus PTK2^down^ (Live, *n*=11), as indicated in Fig. 1A. Gene sets related to cancer modules are presented (**A**, module 98; **B**, module 403; **C**, module 315; **D**, module 125; **E**, module 124; **F**, module 392; **G**, module 451; **H**, module 158; **I**, module 183; **J**, module 61; **K**, module 337; **L**, module 252; **M**, module 57; **N**, module 388; **O**, module 349; **P**, module 198; **Q**, module 244). NES, nominal P-value, and FDR q-values are indicated in the inner panel.


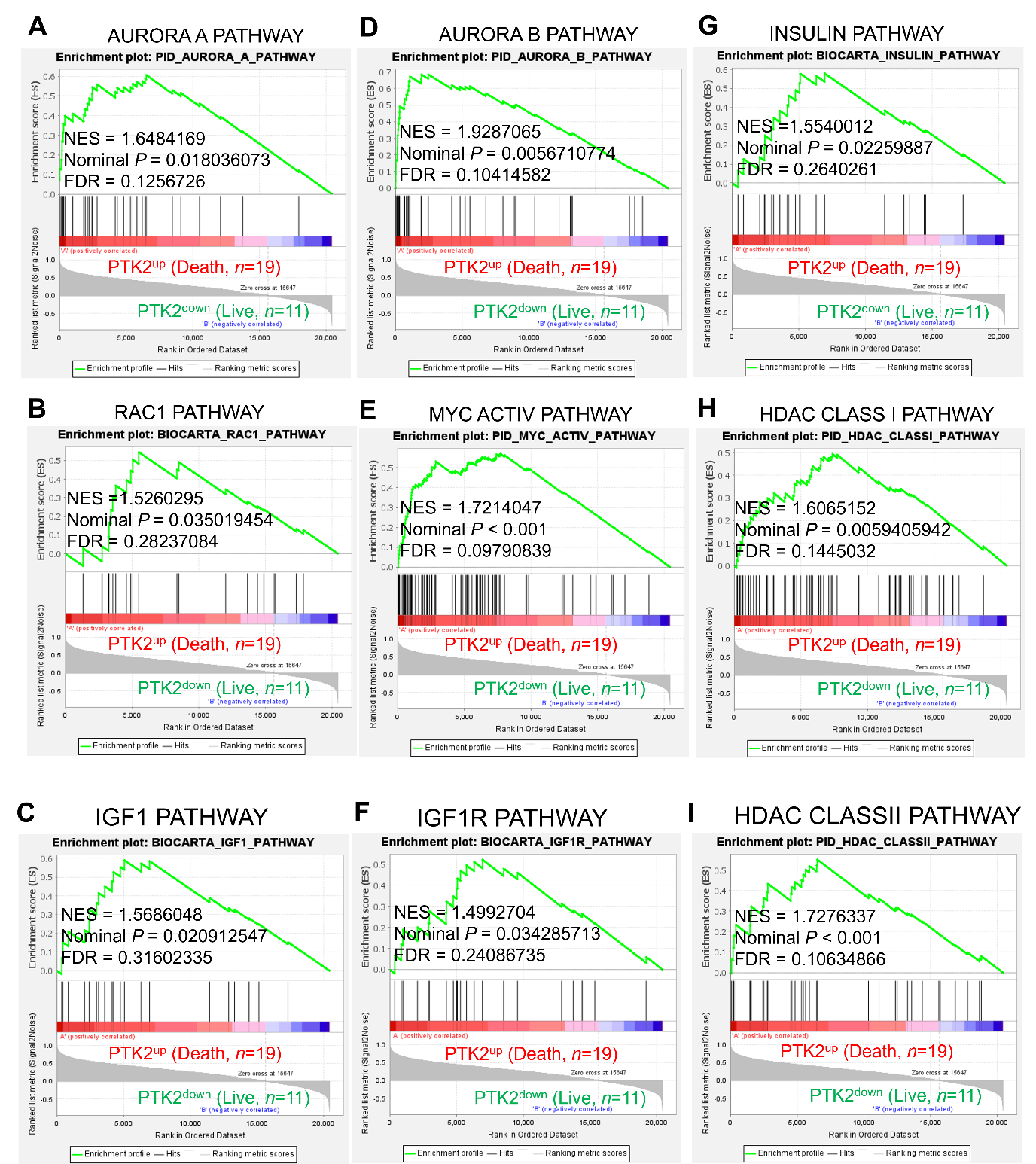


**Fig. S3 A-I** Gene set enrichment analysis (GSEA, https://www.gsea-msigdb.org/gsea/index.jsp) was performed for PTK2^up^ (Death, *n*=19) versus PTK2^down^ (Live, *n*=11), as indicated in Fig. 1A. Gene sets related to cancer progression are presented (**A**, AURORA A PATHWAY; **B**, RAC1 PATHWAY; **C**, IGF1 PATHWAY; **D**, AURORA B PATHWAY; **E**, MYC ACTIV PATHWAY; **F**, IGF1R PATHWAY; **G**, INSULIN PATHWAY; **H**, HDAC CLASS I PATHWAY; **I**, HDAC CLASSII PATHWAY). NES, nominal P-value, and FDR q-values are indicated in the inner panel.


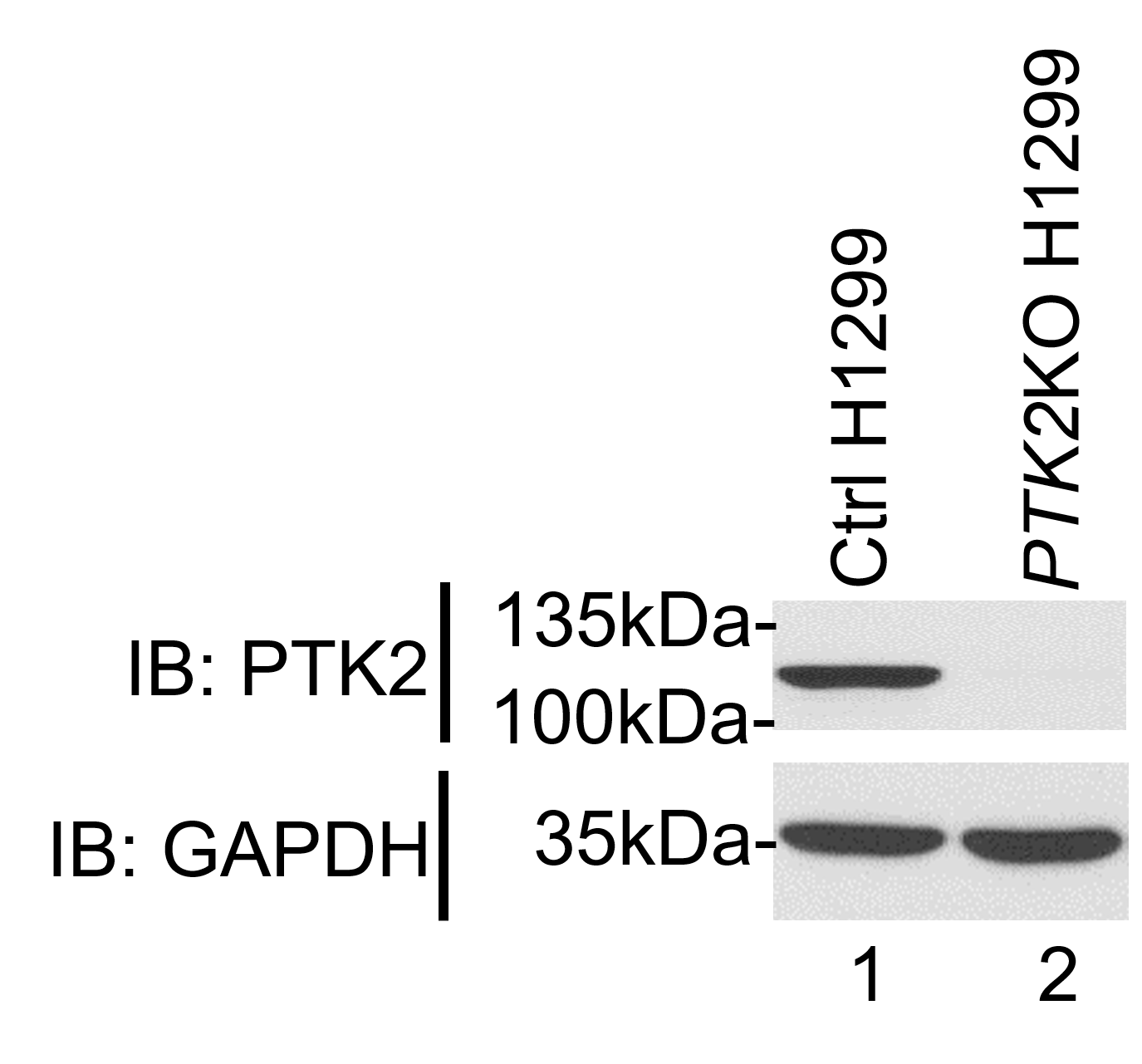


**Fig. S4** Generation of *PTK2*-knockout (*PTK2*-KO) H1299 cells. CRISPR-Cas9 gene editing method Two-vector system was utilized to generate *PTK2*-KO lung cancer cells. Three gRNAs targeted to PTK2 were designed (Fig. 1F). *PTK2*-KO H1299 lung cancer cells were generated using CRISPR-Cas9 gene editing method. Endogenous PTK2 expression was evaluated by western blotting assay with anti-PTK2 and anti-GAPDH antibody (a loading control).


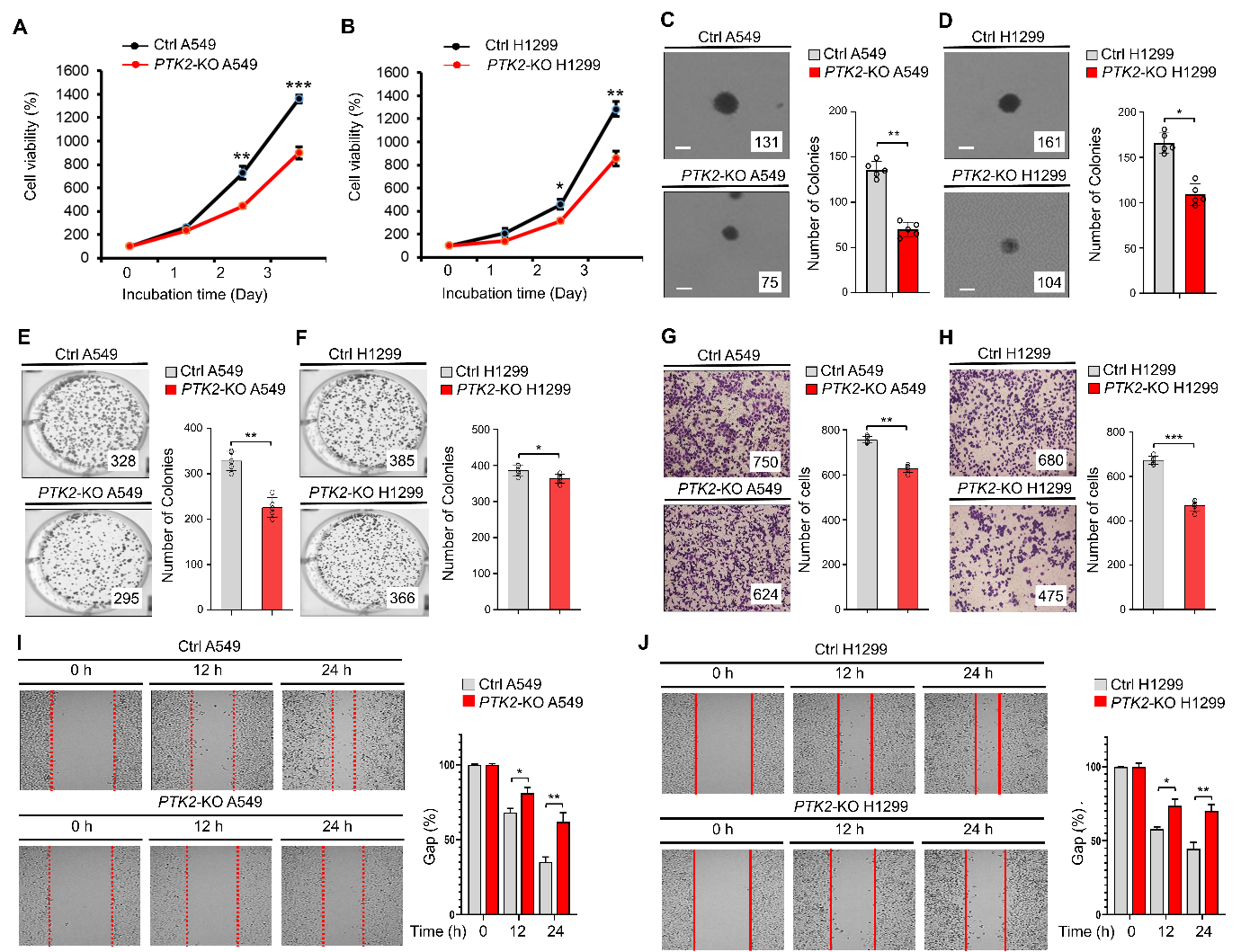


**Fig. S5** Cancer progression assay, including cell proliferation, colony formation, and cell migration. **A** **and B** Cell proliferation assay was performed with control (Ctrl) A549 and *PTK2*-KO A549 cells (**A**) or Ctrl H1299 and *PTK2*-KO H1299 cells (**B**). Results are presented as mean ± SD of three independent experiments. **C and D** Anchorage-independent colony formation assay was performed with Ctrl A549 and *PTK2*-KO A549 cells (**C**) or Ctrl H1299 and *PTK2*-KO H1299 cells (**D**). The number of colonies was counted (scale bar, 100 μm). Results are presented as mean ± SD of five independent experiments. **E and F** Anchorage-dependent colony formation assay was performed with Ctrl A549 and *PTK2*-KO A549 cells (**E**) or Ctrl H1299 and *PTK2*-KO H1299 cells (**F**). The number of colonies was counted. Results are presented as mean ± SD of five independent experiments. **G and H** Transwell migration assay was performed with Ctrl A549 and *PTK2*-KO A549 cells (**G**) or Ctrl H1299 and *PTK2*-KO H1299 cells (**H**). The number of cells was counted. Results are presented as mean ± SD of five independent experiments. **I and J** Wound healing assay was performed with Ctrl A549 and *PTK2*-KO A549 cells (**I**) or Ctrl H1299 and *PTK2*-KO H1299 cells (**J**). The residual gap between migrating cells from the opposing wound edge is expressed as a percentage of initially scraped area (± SD, *n* = 3 different plates). *, *P* < 0.05; **, *P* < 0.01; ***, *P* < 0.001.


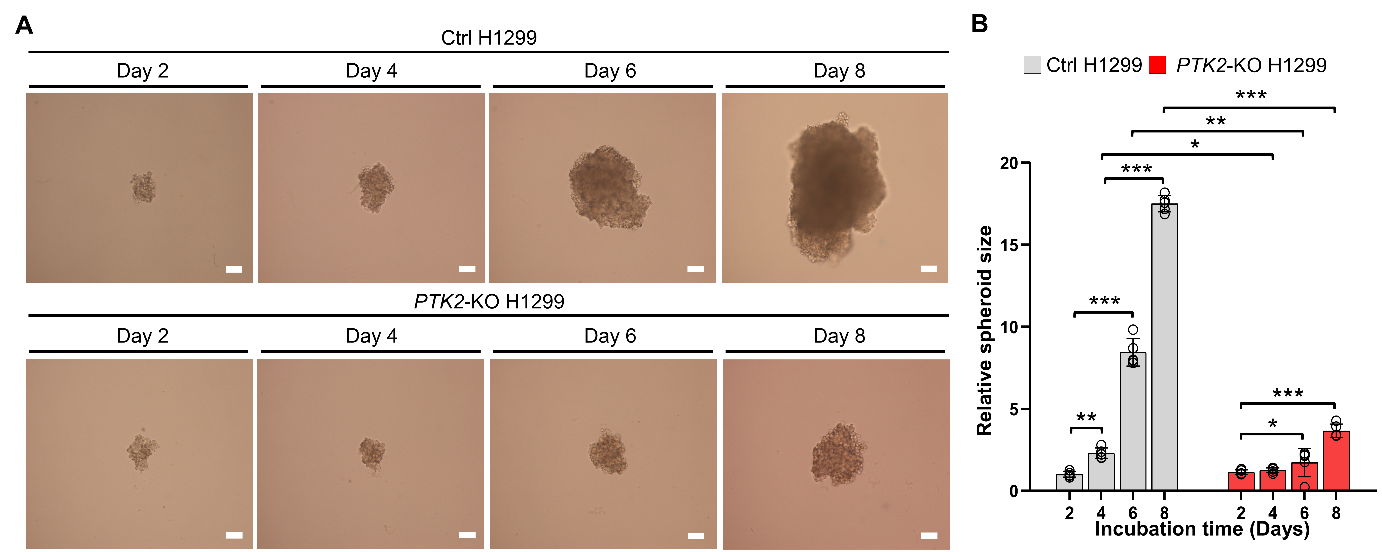


**Fig. S6** 3D tumor spheroid formation assay in Ctrl H1299 and *PTK2*-KO H1299 cells. **A and B** Control (Ctrl) H1299 or *PTK2*-KO H1299 cells in 100 µl growth medium were seeded at a density of 125 cells per well. Plates were incubated at 37 °C for an additional 48 hours to allow the formation of 3D spheroids in culture. The spheroid was incubated for different time periods as indicated. Spheroid formation and growth were evaluated using phase-contrast microscopy (scale bar, 100 μm) (**A**). The size of the spheroid was assessed using ImageJ Software. Error bars represent SD (*n* = 5) of three experiments (**B**). *, *P* < 0.05; **, *P* < 0.01; ***, *P* < 0.001.


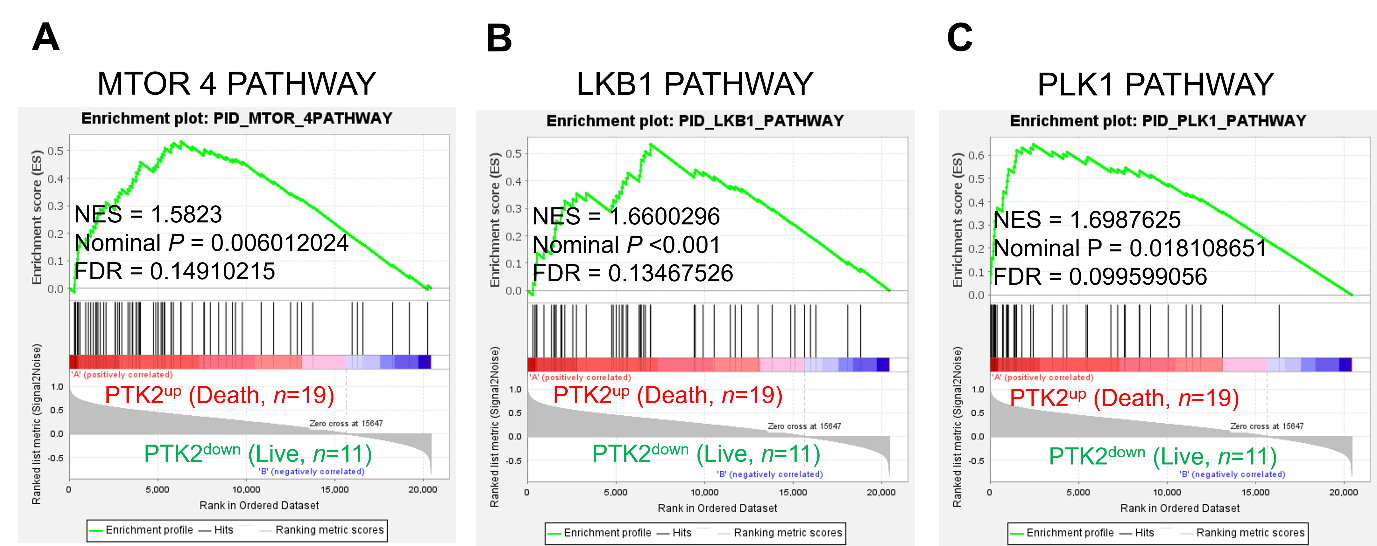


**Fig. S7 A-C** Gene set enrichment analysis (GSEA, https://www.gsea-msigdb.org/gsea/index.jsp) was performed for PTK2^up^ (Death, *n*=19) versus PTK2^down^ (Live, *n*=11), as indicated in Fig. 1A. Gene sets related to EGFR-mediated signaling are presented (**A**, MTOR 4 PATHWAY; **B**, LKB1 PATHWAY; **C**, PLK1 PATHWAY). NES, nominal P-value, and FDR q-values are indicated in the inner panel.


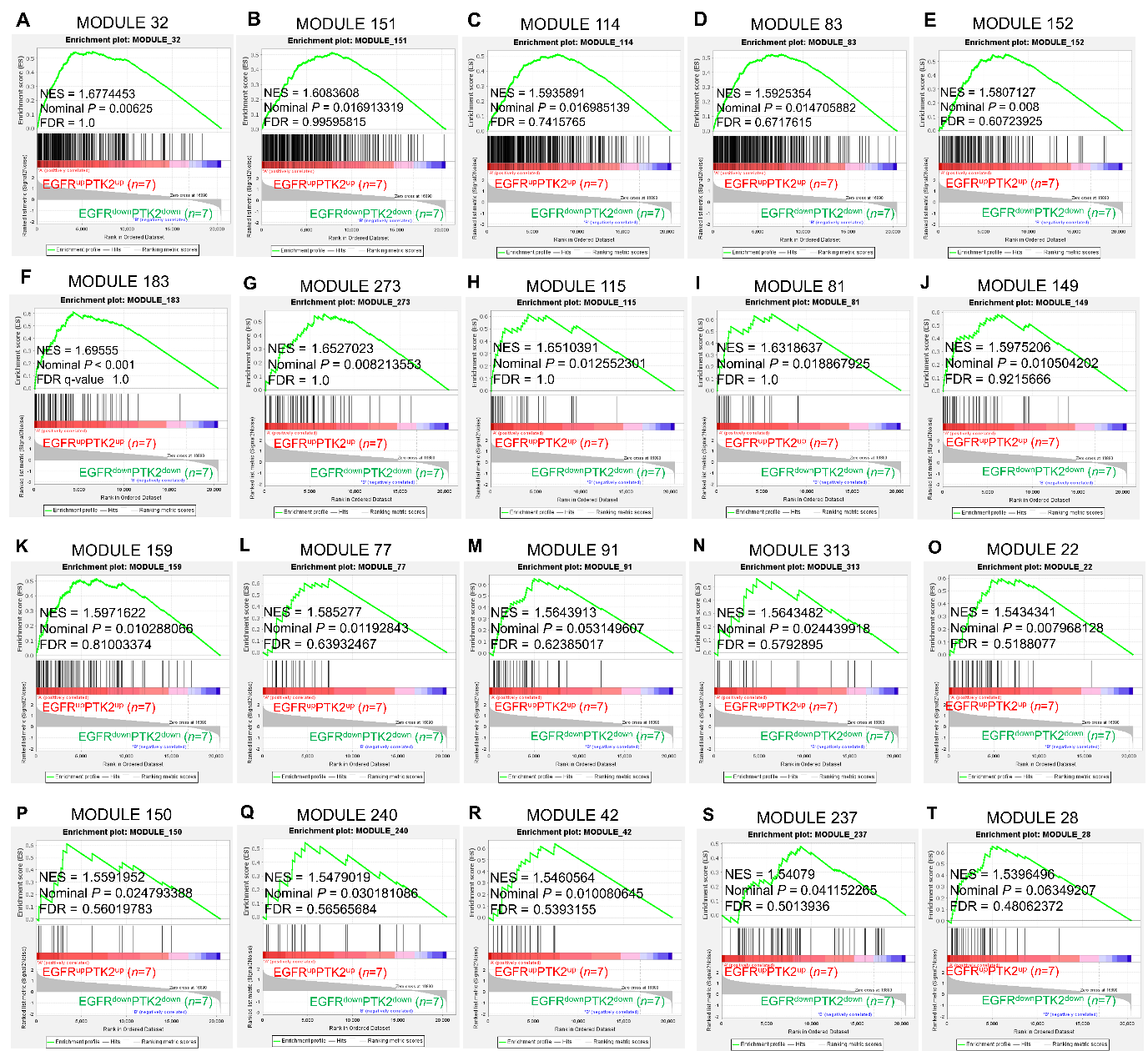


**Fig. S8 A-T** Gene set enrichment analysis (GSEA, https://www.gsea-msigdb.org/gsea/index.jsp) was performed for EGFR^up^PTK2^up^ (*n*=7) versus EGFR^down^PTK2^down^ (*n*=7), as indicated in Fig. 1N. Gene sets related to cancer modules are presented (**A**, module 32; **B**, module 151; **C**, module 114; **D**, module 83; **E**, module 152; **F**, module 183; **G**, module 273; **H**, module 115; **I**, module 81; **J**, module 149; **K**, module 159; **L**, module 77; **M**, module 91; **N**, module 313; **O**, module 22; **P**, module 150; **Q**, module 240; **R**, module 42; **S**, module 237; **T**, module 28). NES, nominal P-value, and FDR q-values are indicated in the inner panel.


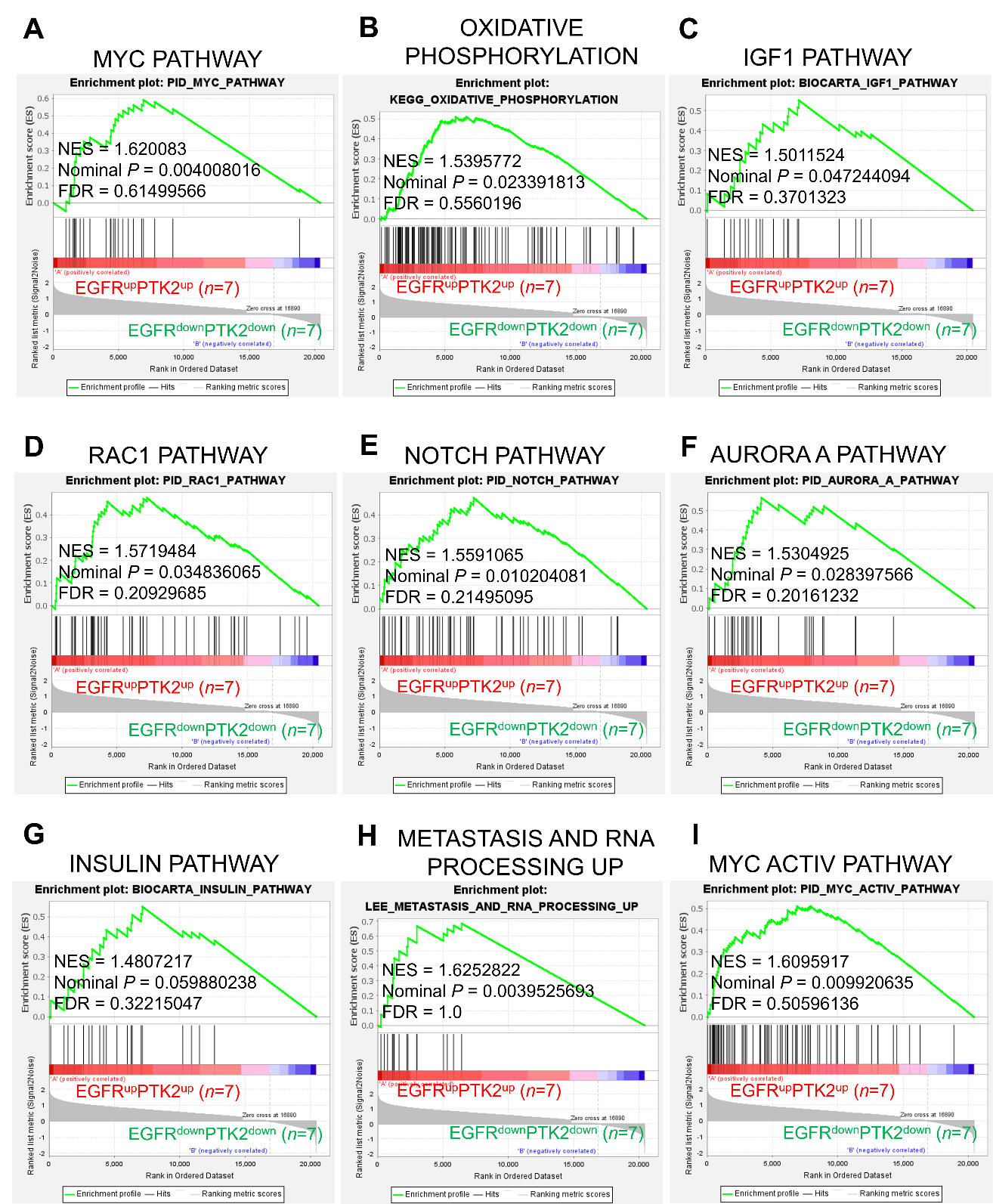


**Fig. S9 A-I** Gene set enrichment analysis (GSEA, https://www.gsea-msigdb.org/gsea/index.jsp) was performed for EGFR^up^PTK2^up^ (*n*=7) versus EGFR^down^PTK2^down^ (*n*=7), as indicated in Fig. 1N. Gene sets related to cancer progression are presented (**A**, MYC PATHWAY; **B**, OXIDATIVE PHOSPHORYLATION; **C**, IGF1 PATHWAY; **D**, RAC1 PATHWAY; **E**, NOTCH PATHWAY; **F**, AURORA A PATHWAY; **G**, INSULIN PATHWAY; **H**, METASTASIS AND RNA PROCESSING UP; **I**, MYC ACTIV PATHWAY). NES, nominal P-value, and FDR q-values are indicated in the inner panel.


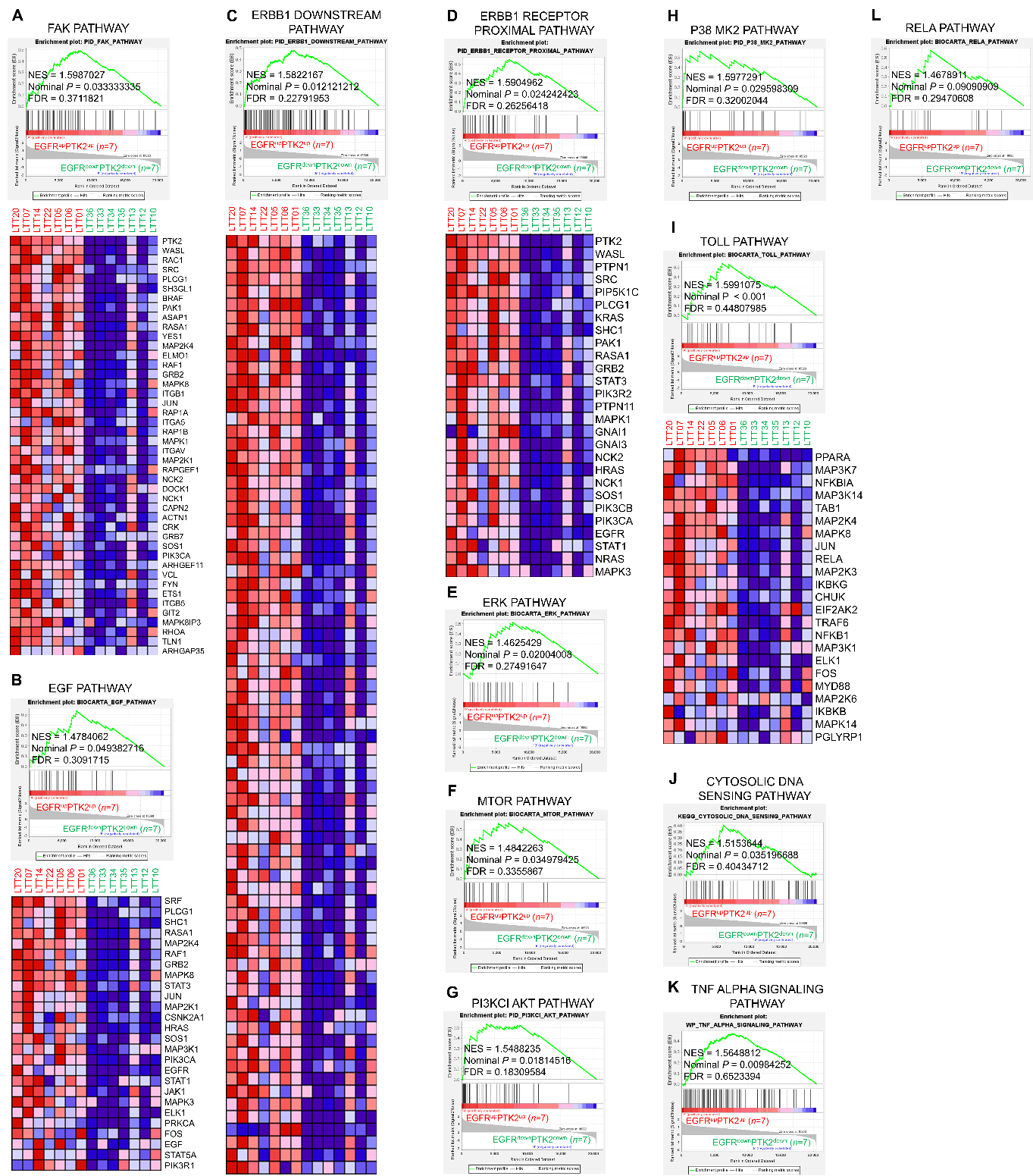


**Fig. S10 A-H** Gene set enrichment analysis (GSEA, https://www.gsea-msigdb.org/gsea/index.jsp) was performed for EGFR^up^PTK2^up^ (*n*=7) versus EGFR^down^PTK2^down^ (*n*=7), as indicated in Fig. 1N. Gene sets related to EGFR-related signaling pathway are presented (**A**, FAK pathway; **B**, EGF pathway; **C**, ERBB1 downstream pathway; **D**, ERBB1 receptor proximal pathway; **E**, ERK pathway; **F**, mTOR pathway; **G**, PI3KCI AKT pathway; **H**, p38 MK2 pathway). Gene expression associated with FAK pathway, EGF pathway, ERBB1 downstream pathway, and ERBB1 receptor proximal pathway are presented in the bottom of each panel. NES, nominal P-value, and FDR q-values are indicated in the inner panel. **I-L** GSEA was performed for EGFR^up^PTK2^up^ (*n*=7) versus EGFR^down^PTK2^down^ (*n*=7). Gene sets related to Toll-related signaling pathway are presented (**I**, Toll pathway; **J**, cytosolic DNA sensing pathway; **K**, TNF-alpha signaling pathway; **L**, RELA pathway). Gene expression associated with Toll the pathway is presented in the bottom of the panel. NES, nominal P-value, and FDR q-values are indicated in the inner panel.


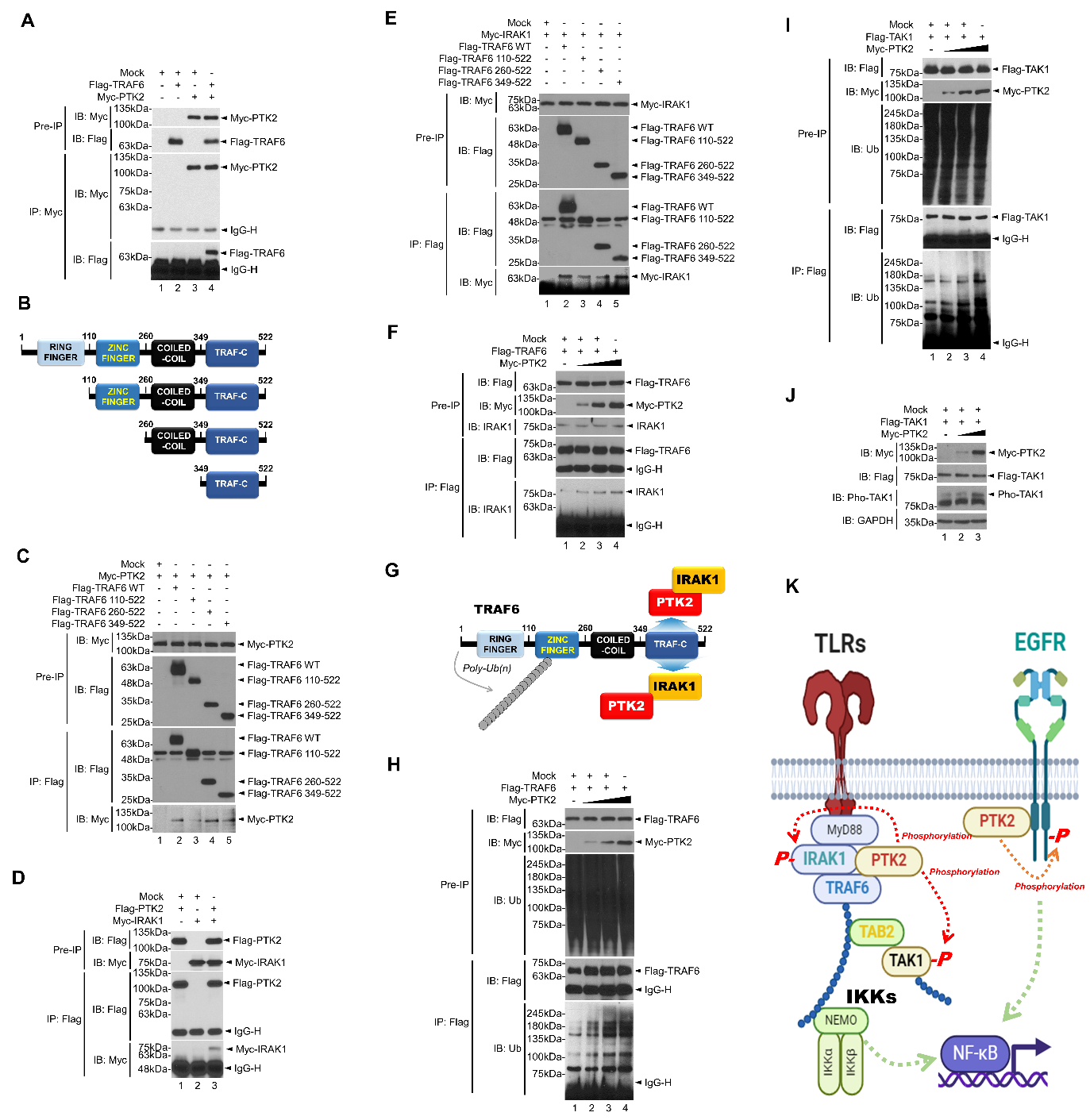


**Fig. S11** Biochemical mechanism study between PTK2 and TLR-mediated signaling. **A** HEK-293T cells were transiently transfected with mock as a control vector, Flag-TRAF6, or Myc-PTK2, as indicated. An immunoprecipitation (IP) assay was performed with anti-Myc antibody. An immunoblotting assay was then performed with anti-Flag or anti-Myc antibody. **B** Truncated mutants of TRAF6 were generated and presented. **C** HEK-293T cells were transiently transfected with mock, Myc-PTK2, or Flag-TRAF6 wild type (WT) and truncated mutants of Flag-TRAF6, as indicated. IP assay was performed with anti-Flag antibody. **D** HEK-293T cells were transiently transfected with mock, Flag-PTK2, or Myc-IRAK1, as indicated. IP assay was performed with anti-Flag antibody. **E** HEK-293T cells were transiently transfected with mock, Myc-IRAK1, or Flag-TRAF6 wild type (WT) and truncated mutants of Flag-TRAF6, as indicated. IP assay was performed with anti-Flag antibody. **F** HEK-293T cells were transiently transfected with mock, Flag-TRAF6, or different concentrations of Myc-PTK2, as indicated. IP assay was performed with anti-Flag antibody and immunoblotting assay was performed with anti-Flag, anti-Myc, and anti-IRAK1 antibodies. **G** A schematic model of how PTK2 enhances the association of TRAF6-IRAK1 complex. **H** H1299 wild type (WT) cells were transiently transfected with mock, Flag-TRAF6, or different concentrations of Myc-PTK2, as indicated. IP assay was performed with anti-Flag antibody and immunoblotting assay was performed with anti-Flag, anti-Myc, and anti-Ub antibodies. **I** H1299 WT cells were transiently transfected with mock, Flag-TAK1, or different concentrations of Myc-PTK2, as indicated. IP assay was performed with anti-Flag antibody and immunoblotting assay was performed with anti-Flag, anti-Myc, and anti-Ub antibodies. **J** A549 WT cells were transiently transfected with mock, Flag-TAK1, or different concentrations of Myc-PTK2, as indicated. Immunoblotting assay was performed with anti-Flag, anti-Myc, anti-pho-TAK1, and anti-GAPDH antibodies. **K** A schematic model of how PTK2 regulates TLR- and EGFR-mediated signaling for NF-κB activation.


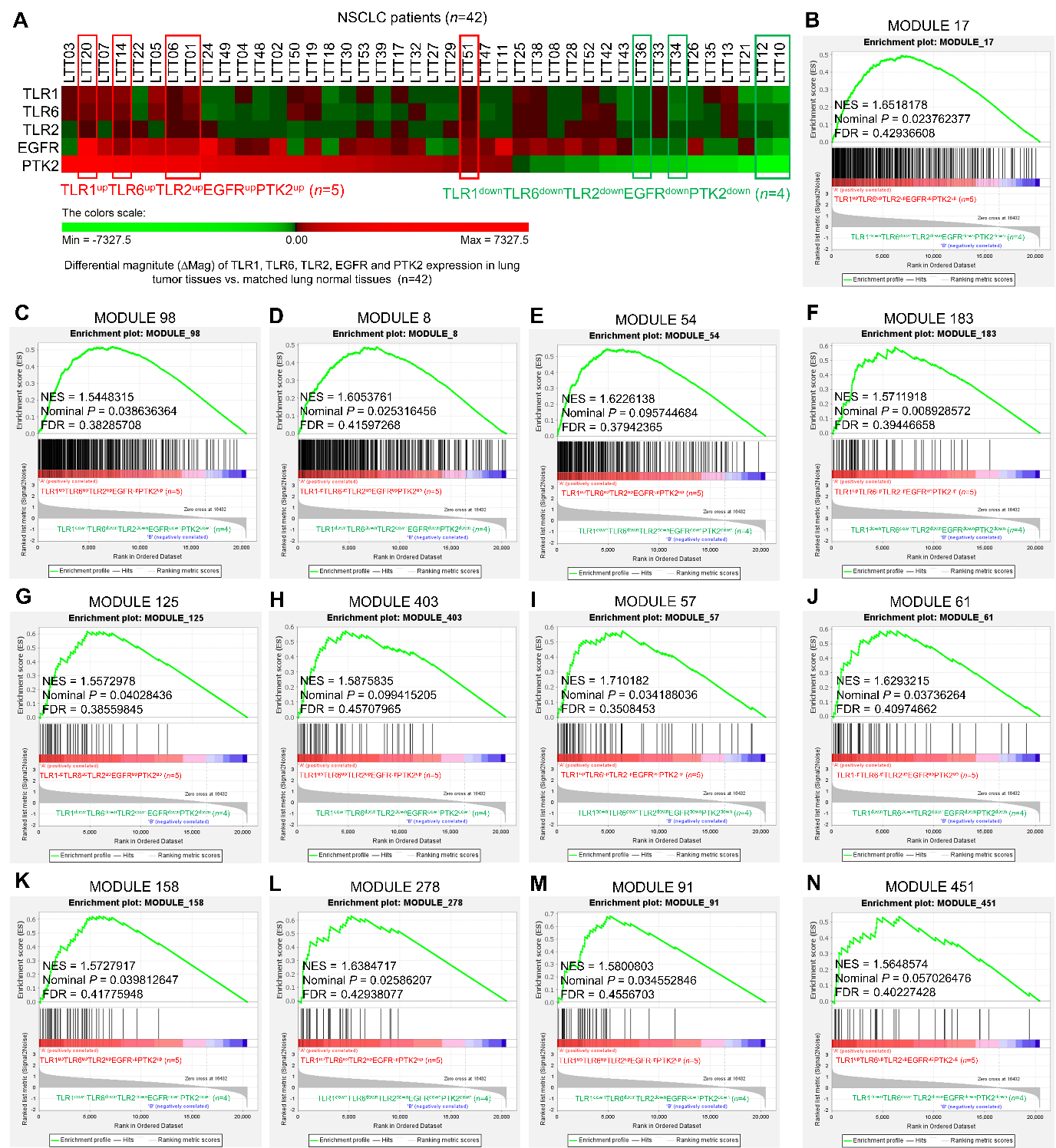


**Fig. S12** Gene set enrichment analysis (GSEA, https://www.gsea-msigdb.org/gsea/index.jsp) was performed for TLR1^up^TLR6^up^TLR2^up^EGFR^up^PTK2^up^ (*n*=5) versus TLR1^down^TLR6^down^TLR2^down^EGFR^down^PTK2^down^ (*n*=4). **A** Based on the △Mag of TLR1, TLR6, TLR2, PTK2, and EGFR, 42 NSCLC patients were divided into patients with TLR1^up^TLR6^up^TLR2^up^EGFR^up^PTK2^up^ (*n*=5, red bar) and patients with TLR1^down^TLR6^down^TLR2^down^EGFR^down^PTK2^down^ (*n*=4, green bar). **B-N** GSEA was performed in TLR1^up^TLR6^up^TLR2^up^EGFR^up^PTK2^up^ (*n*=5) versus TLR1^down^TLR6^down^TLR2^down^EGFR^down^PTK2^down^ (*n*=4). Gene sets related to cancer modules are presented (**B**, module 17; **C**, module 98; **D**, module 8; **E**, module 54; **F**, module 183; **G**, module 125; **H**, module 403; **I**, module 57; **J**, module 61; **K**, module 158; **L**, module 278; **M**, module 91; **N**, module 451). NES, nominal P-value, and FDR q-values are indicated in the inner panel.


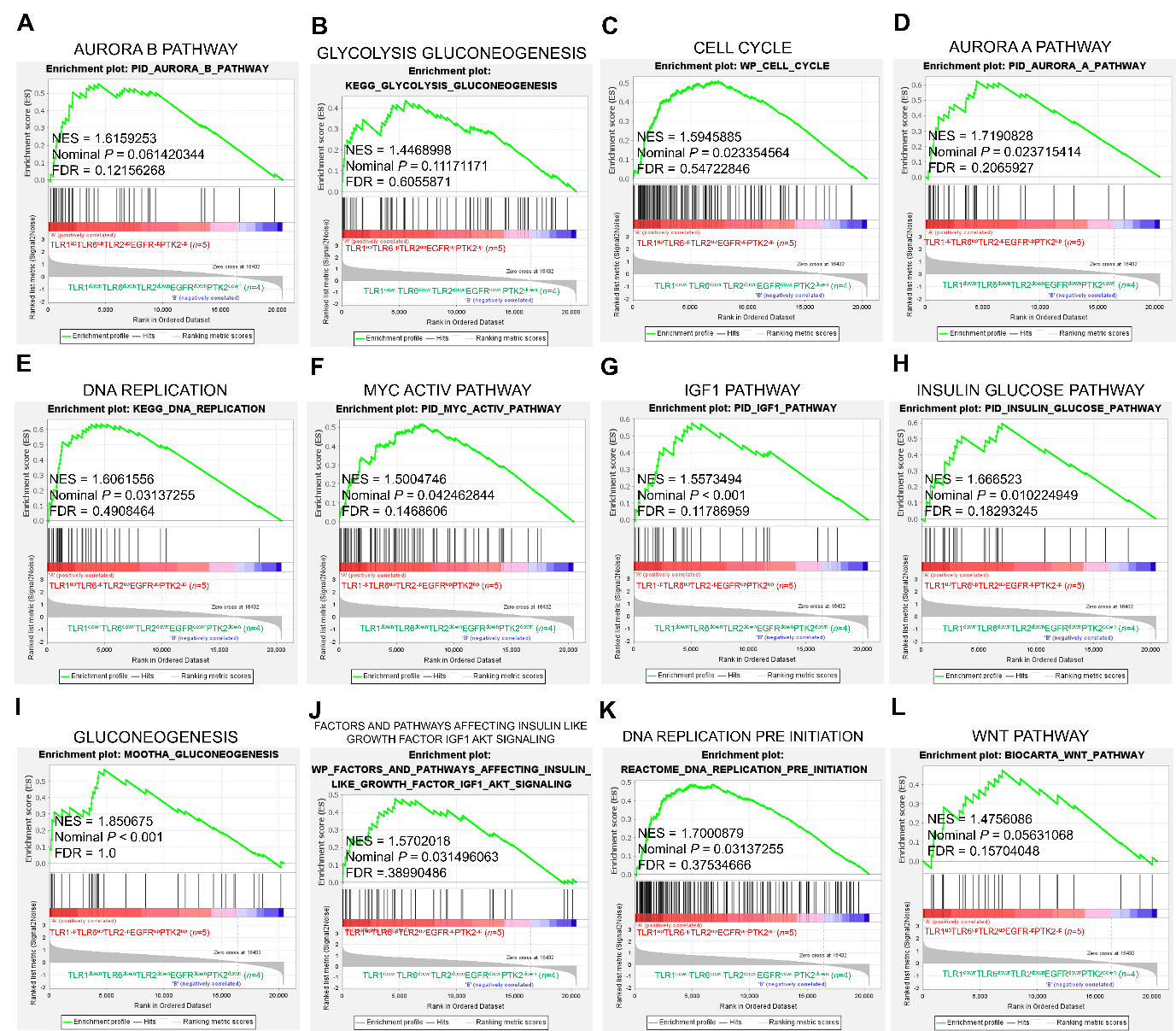


**Fig. S13** Gene set enrichment analysis (GSEA, https://www.gsea-msigdb.org/gsea/index.jsp) was performed in TLR1^up^TLR6^up^TLR2^up^EGFR^up^PTK2^up^ (*n*=5) versus TLR1^down^TLR6^down^TLR2^down^EGFR^down^PTK2^down^ (*n*=4). **A-L** GSEA was performed in TLR1^up^TLR6^up^TLR2^up^EGFR^up^PTK2^up^ (*n*=5) versus TLR1^down^TLR6^down^TLR2^down^EGFR^down^PTK2^down^ (*n*=4). Gene sets related to cancer progression are presented (**A**, AURORA B PATHWAY; **B**, GLYCOLYSIS GLUCONEOGENESIS; **C**, CELL CYCLE; **D**, AURORA A PATHWAY; **E**, DNA REPLICATION; **F**, MYC ACTIV PATHWAY; **G**, IGF1 PATHWAY; **H**, INSULIN GLUCOSE PATHWAY; **I**, GLUCONEOGENESIS; **J**, FACTORS AND PATHWAYS AFFECTING INSULIN LIKE GROWTH FACTOR IGF1 AKT SIGNALING; **K**, DNA REPLICATION PRE INITIATION; **L** WNT PATHWAY). NES, nominal P-value, and FDR q-values are indicated in the inner panel.


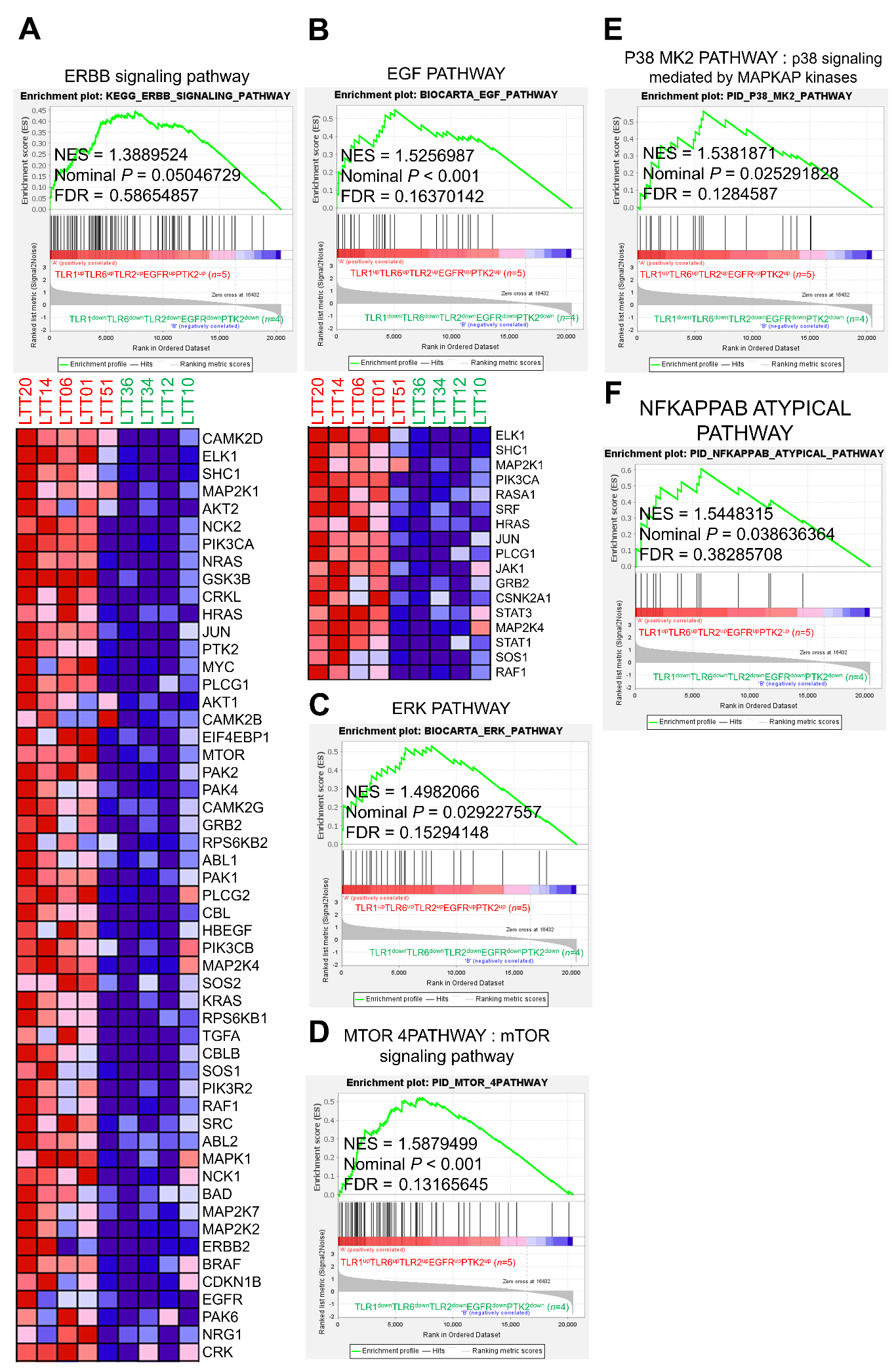


**Fig. S14** Gene set enrichment analysis (GSEA, https://www.gsea-msigdb.org/gsea/index.jsp) was performed in TLR1^up^TLR6^up^TLR2^up^EGFR^up^PTK2^up^ (*n*=5) versus TLR1^down^TLR6^down^TLR2^down^EGFR^down^PTK2^down^ (*n*=4). **A-F** GSEA was performed in TLR1^up^TLR6^up^TLR2^up^EGFR^up^PTK2^up^ (*n*=5) versus TLR1^down^TLR6^down^TLR2^down^EGFR^down^PTK2^down^ (*n*=4). Gene sets related to EGFR-related signaling pathway are presented (**A**, ERBB signaling pathway; **B**, EGF PATHWAY; **C**, ERK PATHWAY; **D**, MTOR 4PATHWAY; **E**, P38 MK2 PATHWAY; **F**, NFKAPPAB ATYPICAL PATHWAY). Gene expression associated with ERBB signaling pathway and EGF pathway are presented in the bottom of each panel. NES, nominal P-value, and FDR q-values are indicated in the inner panel.


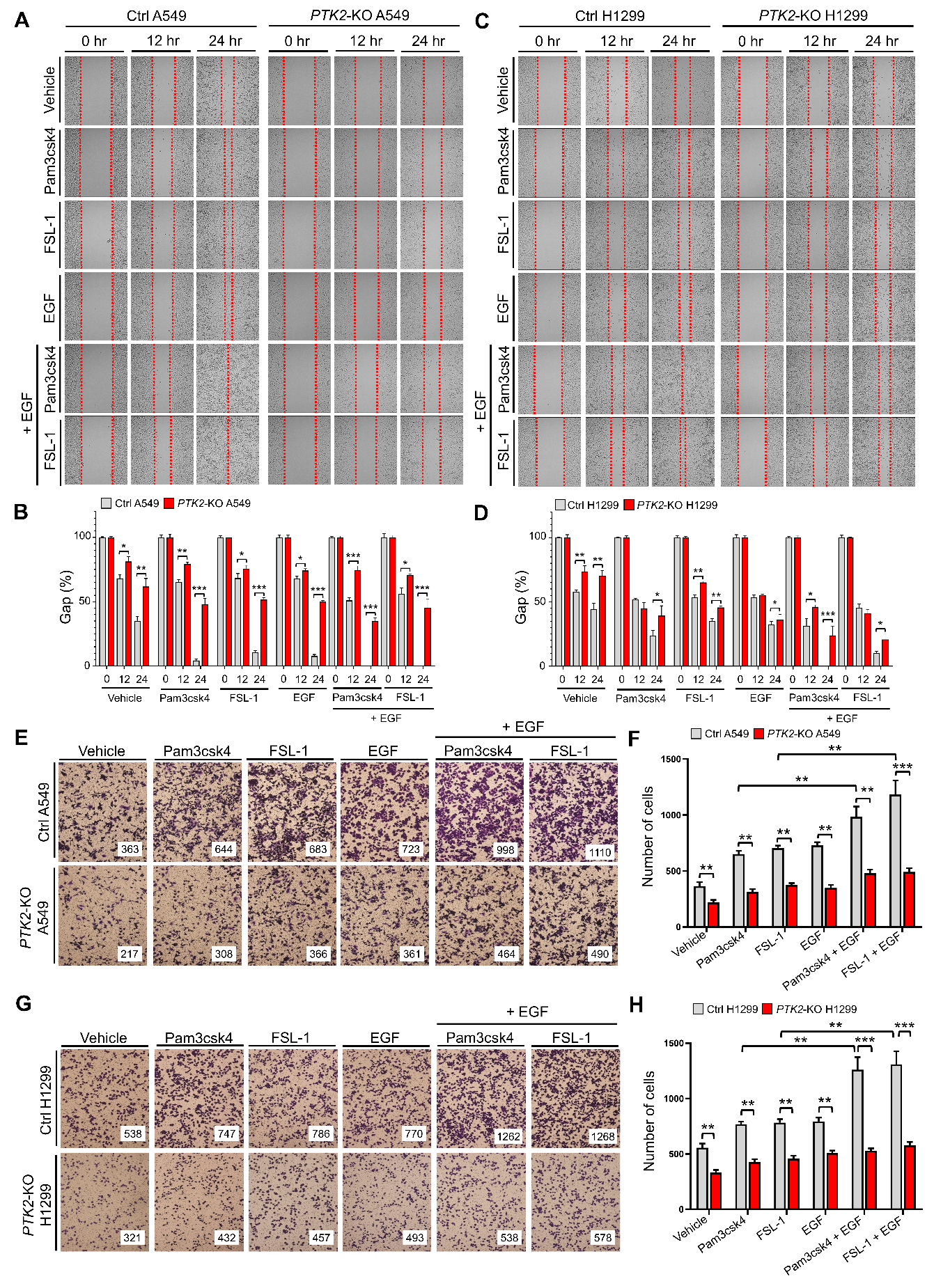


**Fig. S15** *PTK2*-KO lung cancer cells exhibit attenuated cell migration in response to TLR agonists, EGF, or TLR agonist plus EGF stimulation. **A and B** Ctrl A549 and *PTK2*-KO A549 cells were treated with vehicle, Pam3csk4, FSL-1, EGF, Pam3csk4 plus EGF, or FSL-1 plus EGF for different time periods, as indicated. Wound healing assay was performed (**A**). The residual gap between migrating cells from the opposing wound edge is expressed as a percentage of the initial scraped area (± SD, *n* = 3 different plates) (**B**). **C and D** Ctrl H1299 and *PTK2*-KO H1299 cells were treated with vehicle, Pam3csk4, FSL-1, EGF, Pam3csk4 plus EGF, or FSL-1 plus EGF for different time periods, as indicated. Wound healing assay was performed (**C**). The residual gap between migrating cells from the opposing wound edge is expressed as a percentage of the initial scraped area (± SD, *n* = 3 different plates) (**D**). **E and F** Ctrl A549 and *PTK2*-KO A549 cells were treated with vehicle, Pam3csk4, FSL-1, EGF, Pam3csk4 plus EGF, or FSL-1 plus EGF for different time periods, as indicated. Transwell migration assay was performed (**E**). The number of cells was counted. Results are presented as mean ± SD of three independent experiments (**F**). **G and H** Ctrl H1299 and *PTK2*-KO H1299 cells were treated with vehicle, Pam3csk4, FSL-1, EGF, Pam3csk4 plus EGF, or FSL-1 plus EGF for different times, as indicated. Transwell migration assay was performed (**G**). The number of cells was counted. Results are presented as mean ± SD of three independent experiments (**H**). *, P < 0.05; **, P < 0.01; ***, P < 0.001


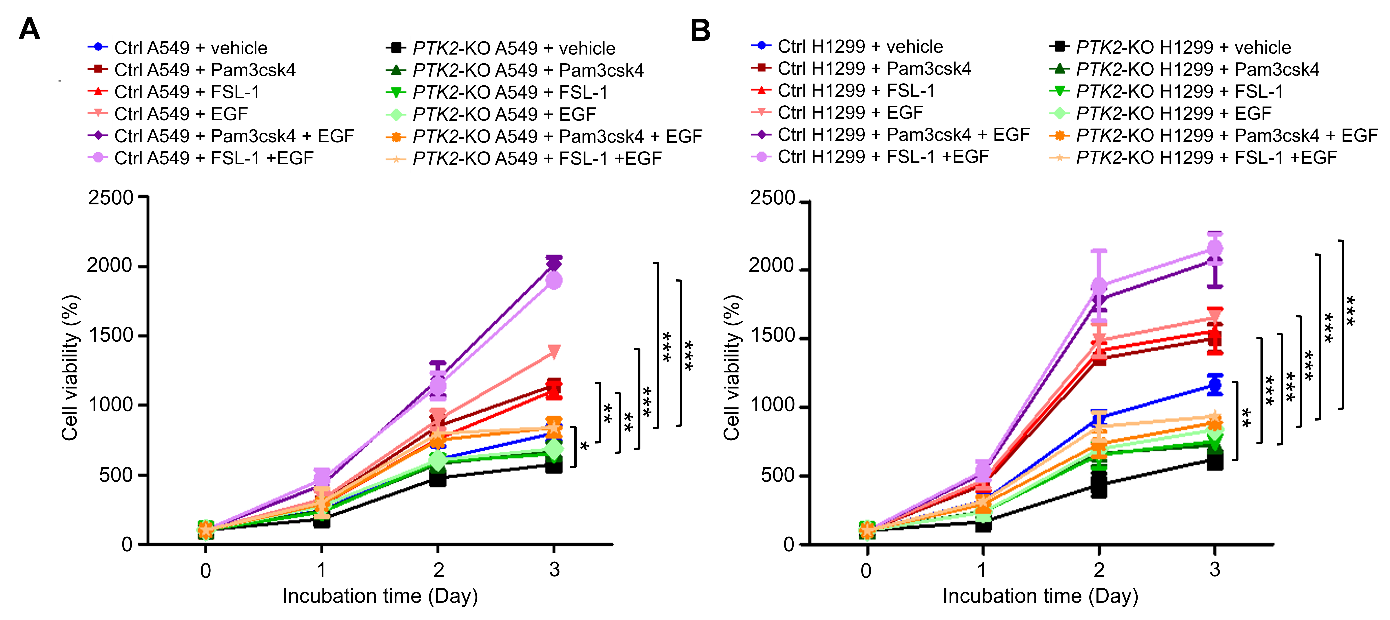


**Fig. S16** Cell proliferation assay. **A** Ctrl A549 and *PTK2*-KO A549 cells were treated with vehicle, Pam3csk4, FSL-1, EGF, Pam3csk4 plus EGF, or FSL-1 plus EGF for different time periods, as indicated. MTT assay was performed. Results are presented as mean ± SD of five independent experiments. **B** Ctrl H1299 and *PTK2*-KO H1299 cells were treated with vehicle, Pam3csk4, FSL-1, EGF, Pam3csk4 plus EGF, or FSL-1 plus EGF for different time periods, as indicated. MTT assay was performed. Results are presented as mean ± SD of five independent experiments. *, *P* < 0.05; **, *P* < 0.01; ***, *P* < 0.001.


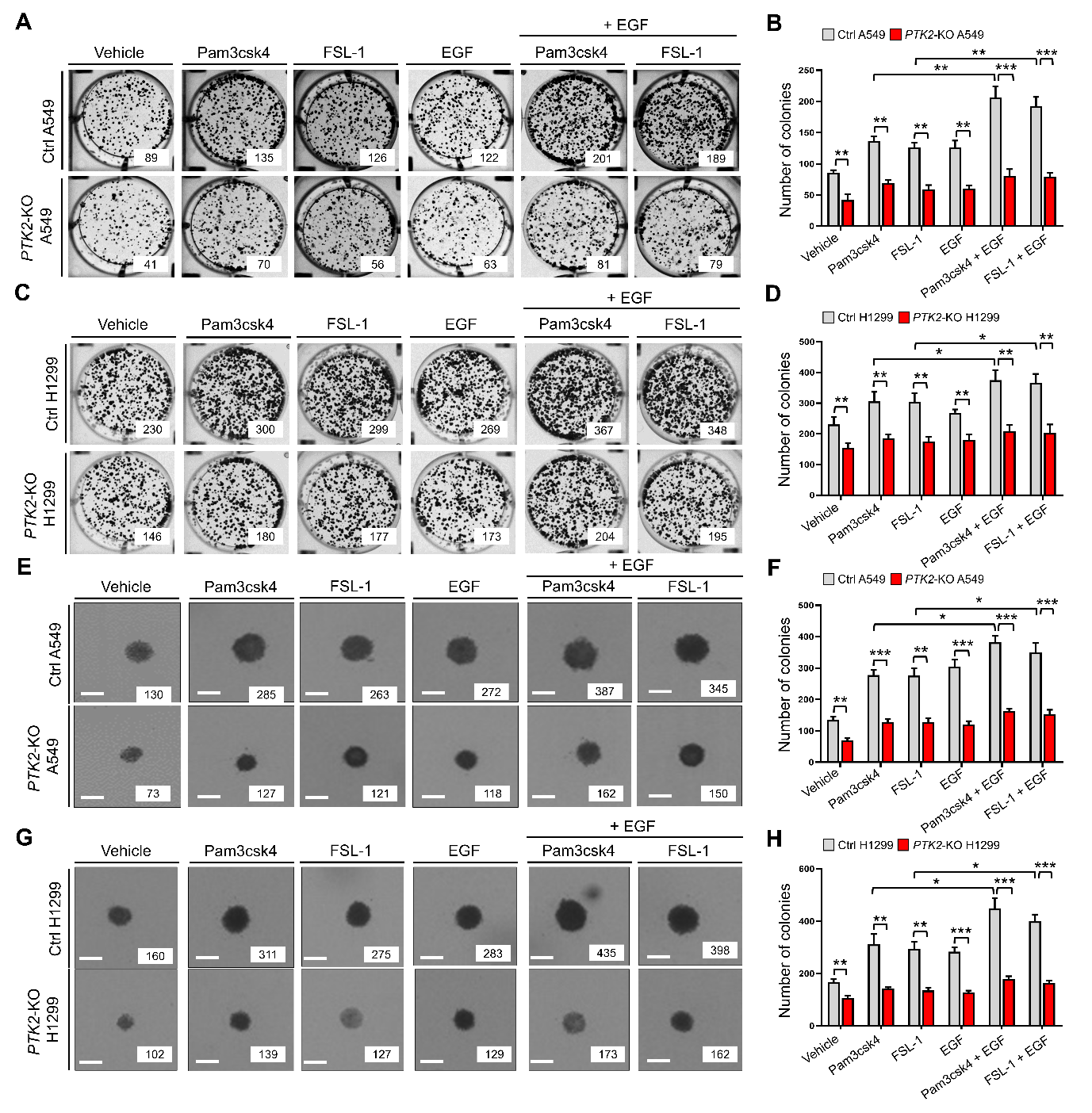


**Fig. S17** *PTK2*-KO lung cancer cells show decreased colony formation induced by TLR agonists, EGF, or TLR agonist plus EGF stimulation. **A-D** Ctrl A549 and *PTK2*-KO A549 (**A** and **B**) or Ctrl H1299 and *PTK2*-KO H1299 (**C** and **D**) cells were treated with vehicle, Pam3csk4, FSL-1, EGF, Pam3csk4 plus EGF, or FSL-1 plus EGF for different time periods, as indicated. Anchorage-dependent colony formation assay was performed. The number of colonies was counted. Results are presented as mean ± SD of three independent experiments. **E-H** Ctrl A549 and *PTK2*-KO A549 (**E** and **F**) or Ctrl H1299 and *PTK2*-KO H1299 (**G** and **H**) cells were treated with vehicle, Pam3csk4, FSL-1, EGF, Pam3csk4 plus EGF, or FSL-1 plus EGF for different time periods, as indicated. Anchorage-independent colony formation assay was performed. The number of colonies was counted. Results are presented as mean ± SD of three independent experiments. *, P < 0.05; **, P < 0.01; ***, P < 0.001.


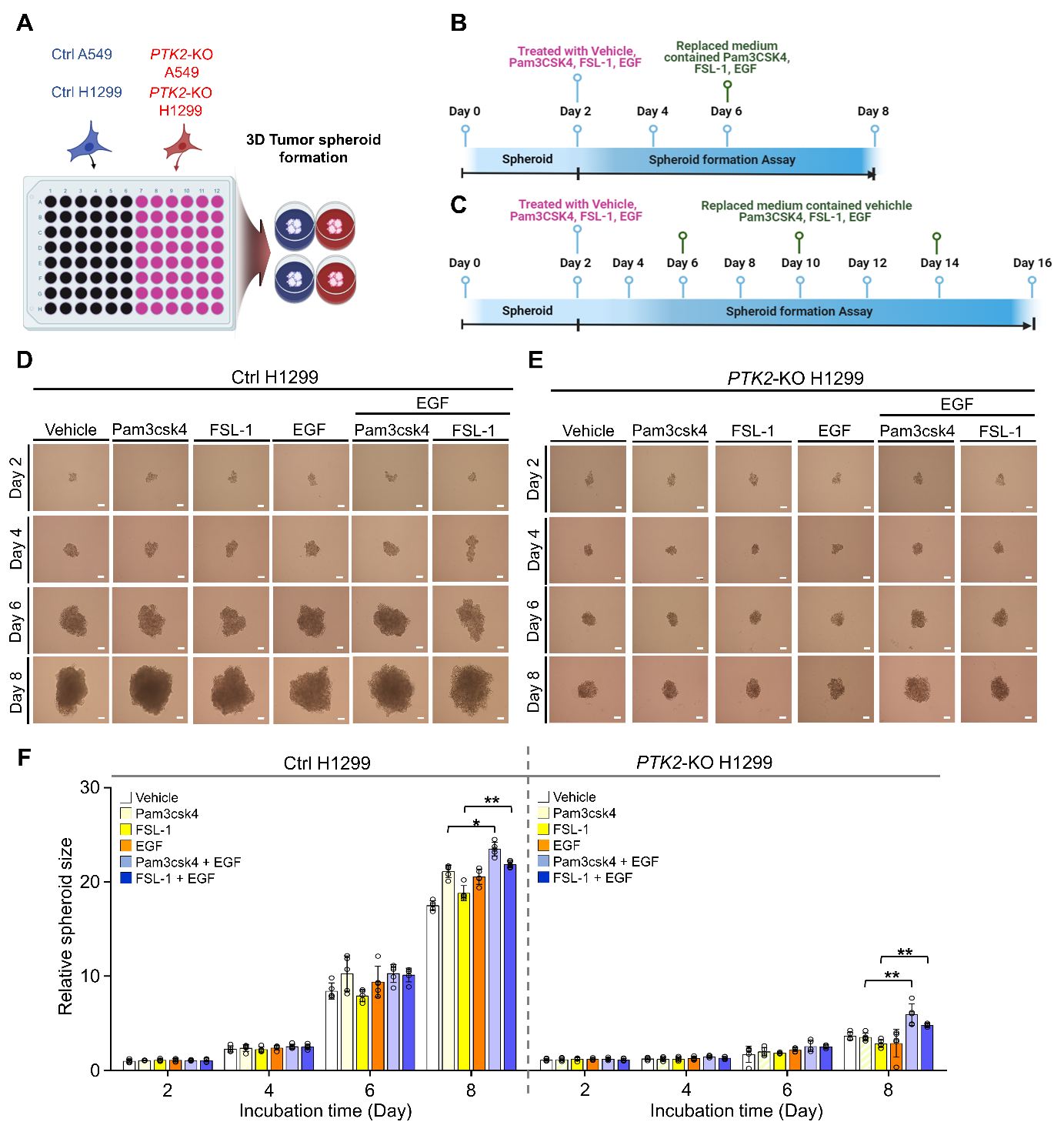


**Fig. S18** 3D tumor spheroid forming activity is decreased in *PTK2*-KO H1299 cells in response to TLR agonists, EGF, or TLR agonist plus EGF stimulation. **A** A schematic view of the experimental set for the 3D tumor spheroid formation assay. Ctrl A549 and *PTK2*-KO A549 or Ctrl H1299 and *PTK2*-KO H1299 cells were seeded into 96-well agarose-hydrogel plates and incubated for 2 days before treatments with TLRs agonists or EGF. **B and C** Experimental schedule for 3D tumor spheroid formation assay in Ctrl H1299 and *PTK2*-KO H1299 cells (**B**) or Ctrl A549 and *PTK2*-KO A549 (**C**) cells was showed. **D-F** At post-cultured day 2 of spheroids, spheroids of Ctrl H1299 (**D**) and *PTK2*-KO H1299 (**F**) cells were treated with vehicle, Pam3csk4, FSL-1, EGF, Pam3csk4 plus EGF, or FSL-1 plus EGF for different time periods. Spheroid formation and growth were evaluated using phase-contrast microscopy (scale bar, 100 μm). The size of the spheroid was assessed using ImageJ Software. Error bars represent SD (*n* = 5) of three experiments. *, P < 0.05; **, P < 0.01.


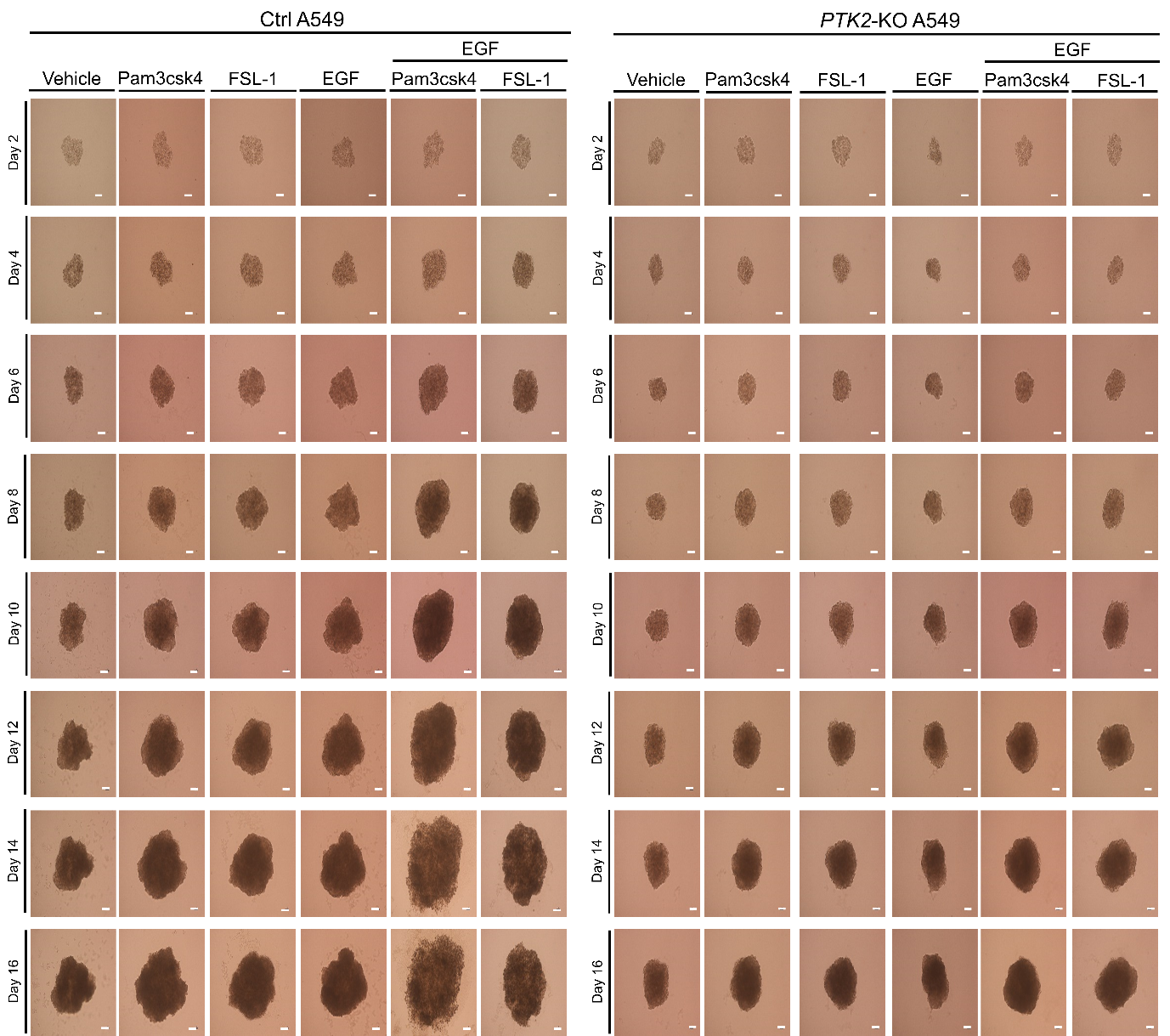


**Fig. S19** Ctrl A549 and *PTK2*-KO A549 cells were seeded into 96-well agarose-hydrogel plates and incubated for 2 days before treatments with TLRs agonists or EGF. At post-cultured day 2 of spheroids, spheroids of Ctrl A549 and *PTK2*-KO A549 cells were treated with vehicle, Pam3csk4, FSL-1, EGF, Pam3csk4 plus EGF, or FSL-1 plus EGF for different time periods. Phase-contrast microscopy (scale bar, 100 μm) images were represented.


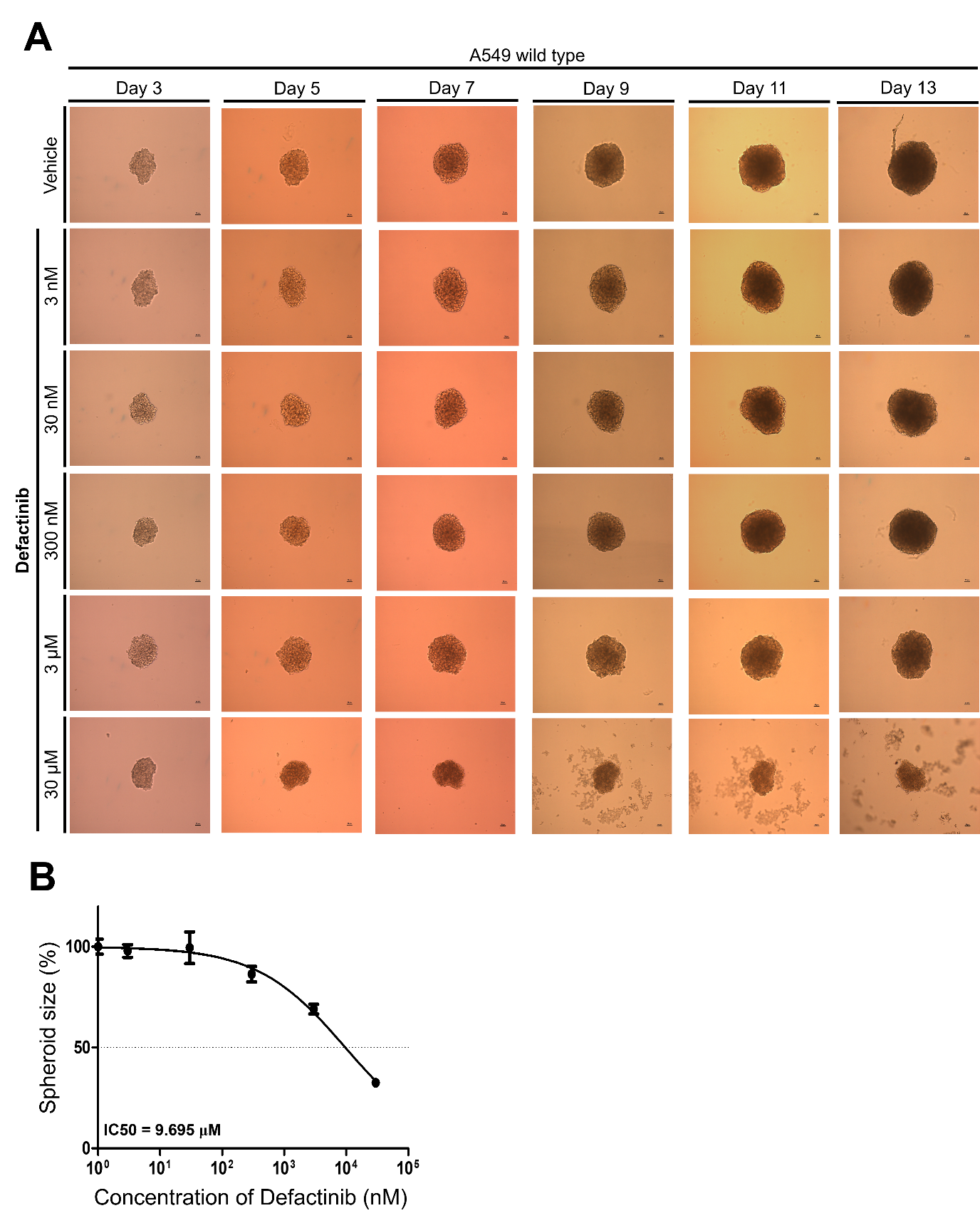


**Fig. S20** The IC50 value of Defactinib in wild type A549 lung cancer cells. **A** Wild type A549 cells were seeded into 96-well agarose-hydrogel plates to generate tumor spheroids and incubated for 2 days before treatments with vehicle or Defactinib. At day 3, tumor spheroids were treated with vehicle or different concentration of Defactinib (3 nM ~ 30 μM), as indicated. 3D tumor spheroid formation assay was performed for different times, as indicated. **B** IC50 value was determined by evaluating spheroid size. Data is represented as mean ± SD of at least three independent experiments (*n*=7, spheroids).


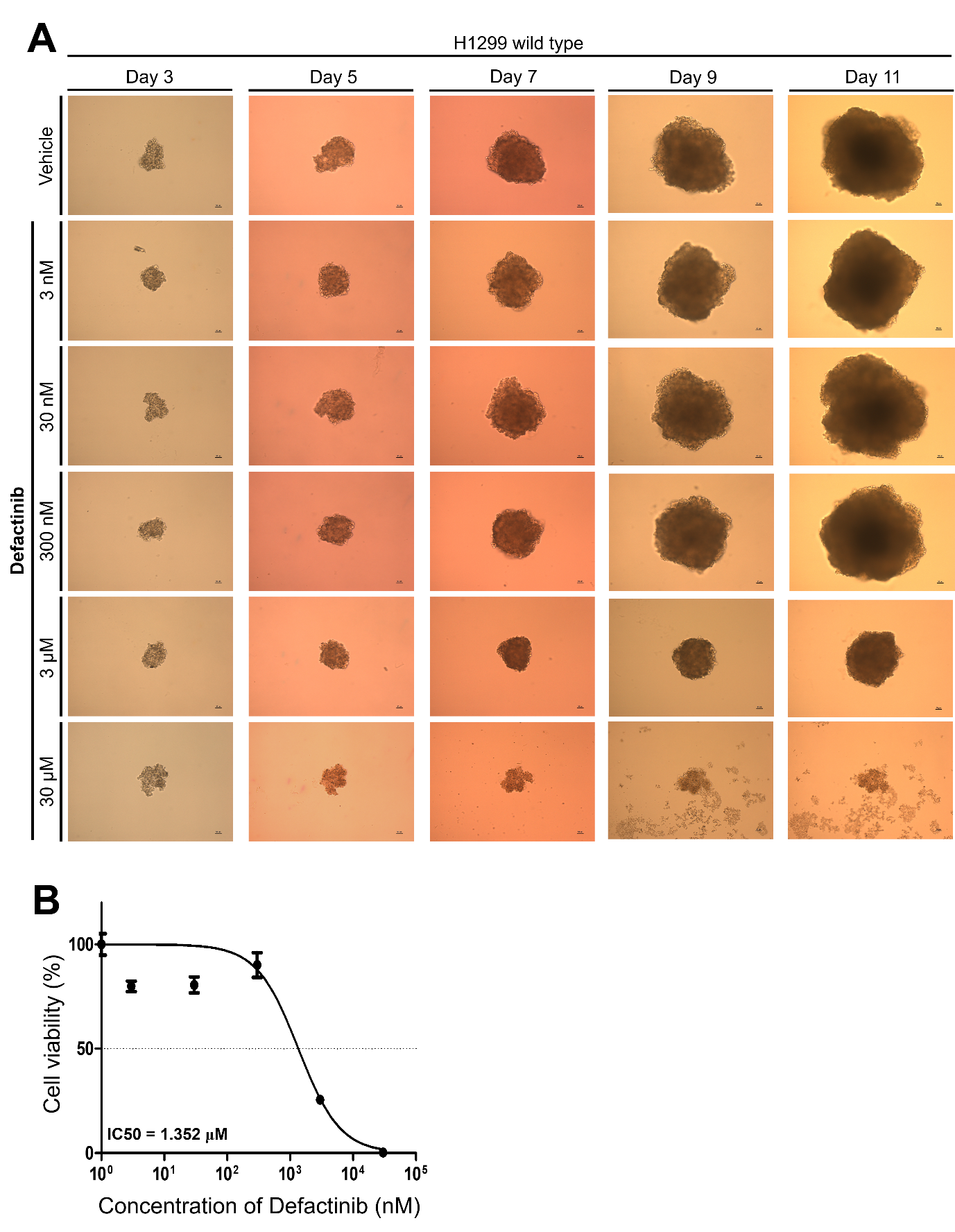


**Fig. S21** The IC50 value of Defactinib in wild type H1299 lung cancer cells. **A** Wild type H1299 cells were seeded into 96-well agarose-hydrogel plates to generate tumor spheroids and incubated for 2 days before treatments with vehicle or Defactinib. At day 3, tumor spheroids were treated with vehicle or different concentration of Defactinib (3 nM ~ 30 μM), as indicated. 3D tumor spheroid formation assay was performed for different times, as indicated. **B** IC50 value was determined by evaluating spheroid size. Data is represented as mean ± SD of at least three independent experiments (*n*=7, spheroids).


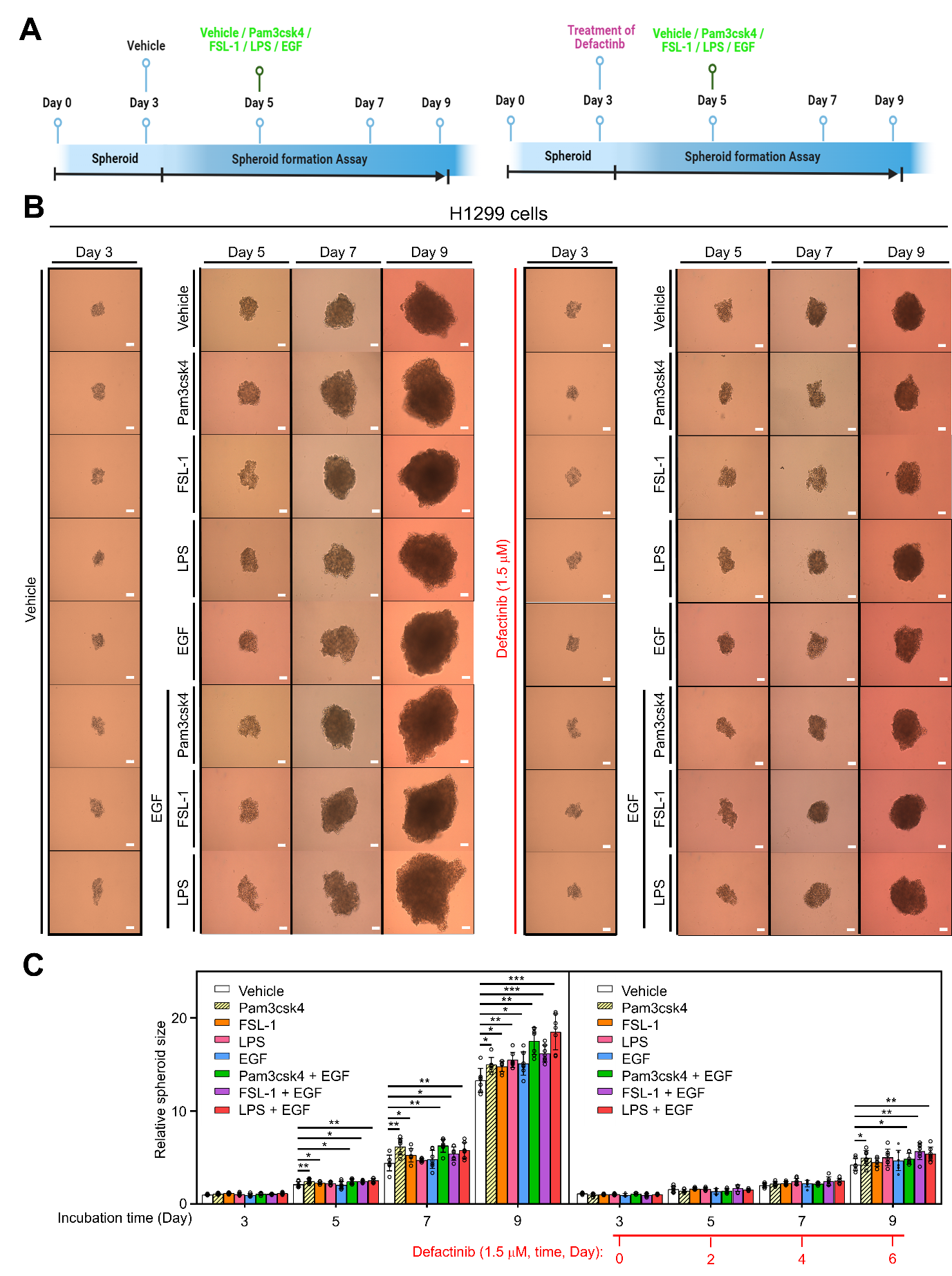


**Fig. S22** Defactinib inhibits 3D tumor spheroid formation in response to Pam3csk4, FSL-1, LPS, EGF, Pam3csk4 plus EGF, FSL-1 plus EGF, or LPS plus EGF. **A** Experimental schedule for 3D tumor spheroid formation assay in wild type H1299 cells. **B** Wild type H1299 cells were seeded into 96-well agarose-hydrogel plates to generate tumor spheroids and incubated for 2 days before treatments with vehicle, Pam3csk4, FSL-1, LPS, EGF, Pam3csk4 plus EGF, FSL-1 plus EGF, LPS plus EGF, or Defactinib. At day 3, tumor spheroids were treated with vehicle, Pam3csk4, FSL-1, LPS, EGF, Pam3csk4 plus EGF, FSL-1 plus EGF, and LPS plus EGF in the presence or absence of Defactinib, as indicated. 3D tumor spheroid formation assay was performed for different times, as indicated. **C** The size of the spheroid was assessed using ImageJ Software. Error bars represent SD (*n* = 5) of three experiments. *, *P* < 0.05; **, *P* < 0.01; ***, *P* < 0.001.


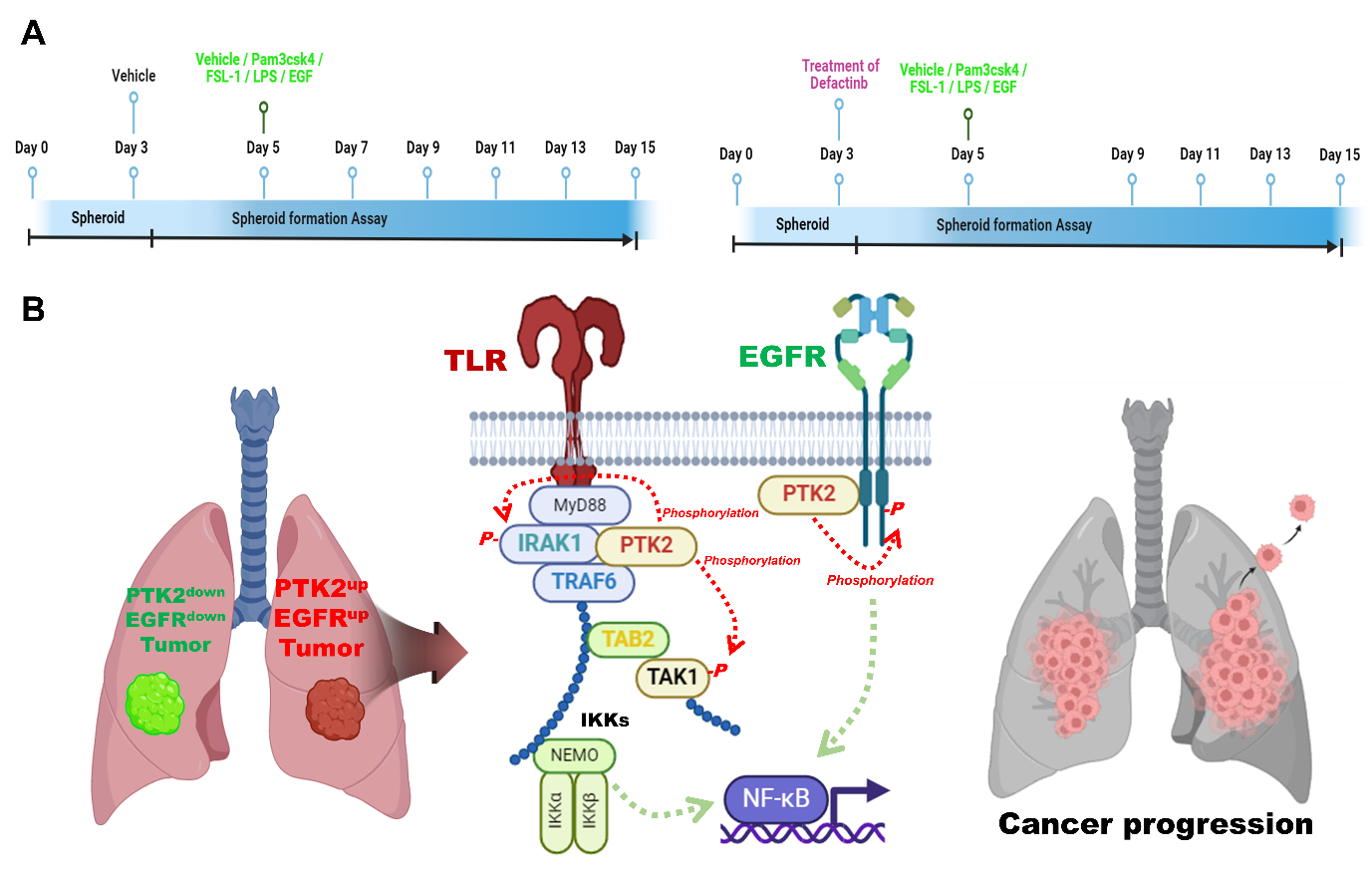


**Fig. S23 A** Experimental schedule for 3D tumor spheroid formation assay in wild type A549 cells. **B** A possible model of how PTK2 promotes lung cancer progression in PTK2^up^EGFR^up^ patients via the cross-talk between EGFR- and TLR-mediated signaling. NSCLC patients with up-regulated PTK2 and EGFR are more likely to show lung cancer progression induced by EGFR and TLRs than NSCLC patients with down-regulated PTK2 and EGFR (**left**). In terms of mechanical aspects, PTK2 induces activation of TLR-medicated signaling molecules, such as IRAK1, TRAF6, and TAK1, and simultaneously induces activation of EGFR, consequently leading to activation of NF-κB (**middle**). These signaling events eventually promote lung cancer progression (**right**).

**Table S1** Clinical characteristics of NSCLC patients and ∆Mag (∆PTK2) of PTK2 in NSCLC patients (*n*=42).

**Table S2** Differential magnitude (Δ Mag) of EGFR and PTK2 expression between lung tumor tissues (LTTs) and matched lung normal tissues (mLNTs) and patient's vital statues.

**Table S3** Differential magnitude (Δ Mag) of EGFR and PTK2 expression between lung tumor tissues (LTTs) and matched lung normal tissues (mLNTs); PTK2^up^EGFR^up^ NSCLC patients (*n*=7) vs. PTK2^down^EGFR^down^ NSCLC patients (*n*=7).

**Table S4** Differential magnitude (Δ Mag) of TLR1, TLR6, TLR2, EGFR, and PTK2 expression between lung tumor tissues (LTTs) and matched lung normal tissues (mLNTs); TLR1^up^TLR6^up^TLR2^up^PTK2^up^EGFR^up^ NSCLC patients (*n*=5) vs. TLR1^down^TLR6^down^TLR2^down^PTK2^down^EGFR^down^ NSCLC patients (*n*=4).
